# In vivo synergistic tumor therapies based on copper sulfide photothermal therapeutic nanoplatforms

**DOI:** 10.1002/EXP.20220161

**Published:** 2023-06-24

**Authors:** Jingwen Ma, Na Li, Jingjian Wang, Zhe Liu, Yulong Han, Yun Zeng

**Affiliations:** ^1^ Radiology Department CT and MRI Room Ninth Hospital of Xi'an Ninth Affiliated Hospital of Medical College of Xi'an Jiaotong University Xi'an Shaanxi Province P. R. China; ^2^ Department of Pathology Ninth Hospital of Xi'an Ninth Affiliated Hospital of Medical College of Xi'an Jiaotong University Xi'an Shaanxi Province P. R. China; ^3^ School of Engineering and Applied Sciences Harvard University Cambridge Massachusetts USA; ^4^ School of Life Science and Technology Xidian University and Engineering Research Center of Molecular and Neuro Imaging, Ministry of Education Xi'an Shaanxi Province P. R. China; ^5^ International Joint Research Center for Advanced Medical Imaging and Intelligent Diagnosis and Treatment and Xi'an Key Laboratory of Intelligent Sensing and Regulation of trans‐Scale Life Information, School of Life Science and Technology Xidian University Xi'an Shaanxi Province P. R. China

**Keywords:** copper sulfide, photothermal therapy, synergistic therapy, tumor treatment

## Abstract

Tumor cells may be eliminated by increasing their temperature. This is achieved via photothermal therapy (PTT) by penetrating the tumor tissue with near‐infrared light and converting light energy into heat using photothermal agents. Copper sulfide nanoparticles (CuS NPs) are commonly used as PTAs in PTT. In this review, we aimed to discuss the synergism between tumor PTT with CuS NPs and other therapies such as chemotherapy, radiotherapy, dynamic therapies (photodynamic, chemodynamic, and sonodynamic therapy), immunotherapy, gene therapy, gas therapy, and magnetic hyperthermia. Furthermore, we summarized the results obtained with a combination of two treatments and at least two therapies, with PTT as one of the included therapies. Finally, we summarized the benefits and drawbacks of various therapeutic options and state of the art CuS‐based PTT and provided future directions for such therapies.

## Graphical abstract

The synergism between tumor photothermal therapy (PTT) with copper sulfide nanoparticles and other therapies is discussed such as chemotherapy, radiotherapy, dynamic therapies (photodynamic, chemodynamic, and sonodynamic therapy), immunotherapy, gene therapy, gas therapy, and magnetic hyperthermia. Further, a combination of two treatments and at least two therapies with PTT is summarized.

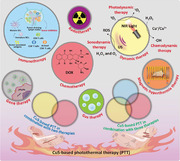

## 1 INTRODUCTION

Malignant tumor seriously threatens human health owing to undesirable characteristics, such as uncontrolled proliferation, aberrant differentiation, invasion, and metastasis.^[^ ^1^ ^]^ The three primary clinical cancer therapies include surgical resection, chemotherapy, and radiotherapy. Surgery is an invasive therapy that cannot eradicate minimal lesions. Moreover, many tumors are inoperable. Chemotherapy and radiotherapy can efficiently eradicate tumors to a certain extent; however, these therapies are associated with significant drawbacks, including the possibility of off‐targeting, drug or radiation resistance, limited specificity, fatigue, discomfort, hair loss, digestive issues, inflammatory responses, and immune system damage.^[^ ^2^, ^3^, ^4^ ^]^ Photothermal therapy (PTT) is a promising therapeutic method that relies on the high near‐infrared (NIR) light absorption of tissue and high photothermal conversion efficiency (PCE) of photothermal agents (PTAs).^[^ ^5^ ^]^ PTT may help augment blood flow,^[^ ^6^ ^]^ improve the uptake of chemotherapeutic drugs into the tumor tissues, and even alleviate tumor hypoxia.^[^ ^7^ ^]^ Conversely, PTAs can convert light into heat to kill tumor cells. High‐power lasers can heat tissues directly but at the expense of affecting healthy tissues. The higher PCE of PTAs suggests that more light energy can be converted to heat; therefore, fewer PTAs can be used. To achieve PTT with a lower‐power laser, nanomaterials with strong photothermal conversion capabilities are often utilized owing to their penetrability and retention ability in tumor tissues.

Gold nanomaterials, inorganic sulfides, graphene, and conjugated polymers are currently employed as PTAs.^[^ ^8^ ^]^ Copper sulfide nanoparticles (CuS NPs) have received considerable attention as a PTA in recent years, and there have been several reports over the last decade.^[^ ^9^, ^10^ ^]^ Many methods of synthesizing CuS NPs have been developed, such as microwave irradiation, thermolysis, hydrothermal/solvothermal method, hot‐injection method, electrodeposition, ion exchange, and template‐free one‐step synthesis.^[^ ^10^ ^]^ The shape and size of NPs are tunable according to these methods. CuS NPs offer high optical qualities and minimal cytotoxicity, and localized surface plasmon resonance (LSPR) contributes to their NIR absorbance, mainly through d‐d transitions of Cu^2+^, without the variations induced by the morphology and dielectric constant of the environment.^[^ ^11^ ^]^ Owing to the lack of Cu atoms in CuS‐based materials, free holes are produced in the valence band, generating a self‐doped p‐type semiconductor. Further, owing to LSPR, CuS NPs display significant NIR absorbance despite their absence of heavy cation (hole‐doped). The LSPR of CuS NPs may convert the light received into thermal energy.^[^ ^12^, ^13^ ^]^ However, the PCE of CuS NPs is low. Accordingly, CuS NPs should be utilized in conjunction with other therapeutic agents to maximize their antitumor efficacy. Nevertheless, their ease of production, improved biological inertness, and maximum absorbance matching NIR wavelength are relevant advantages compared with other nanomaterials. In addition to enhancing tumor suppression, combining other nanomaterials may also increase the biosafety of CuS NPs. CuS NPs with smaller sizes can bypass the renal threshold and will be quickly metabolized and excreted by the kidneys, penetrate deeply into tumor locations, and are less expensive than gold nanomaterials.^[^ ^14^ ^]^ Therefore, during the last five years, CuS NP‐based PTT has been widely employed in conjunction with other medicines to treat cancers.

**SCHEME 1:**
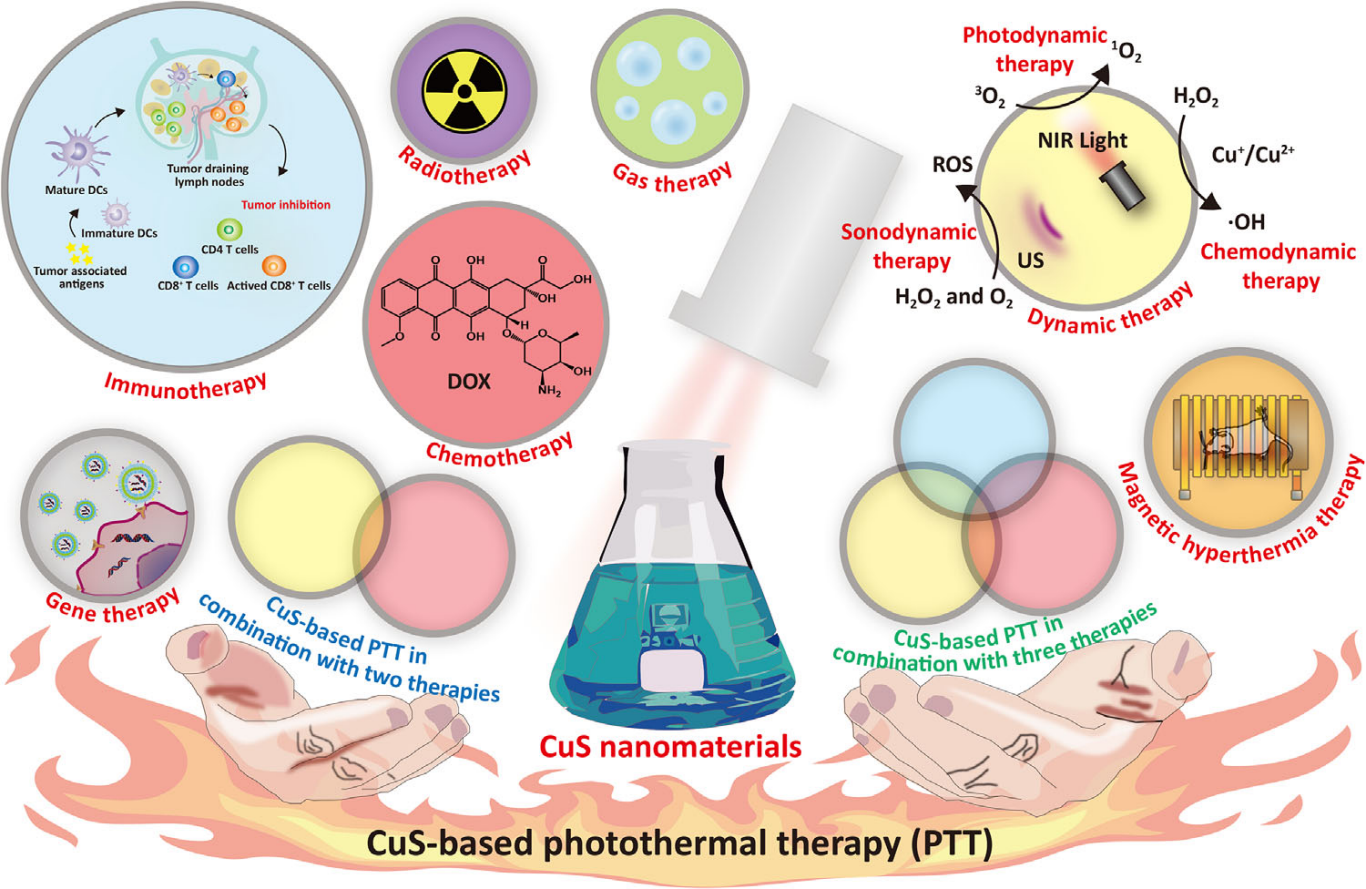
Schematic illustration of copper sulfide (CuS)‐based photothermal therapy (PTT) combined with tumor therapeutic strategies.

In this review, we aimed to discuss the utilization of CuS NPs in tumor PTT over the past 5 years (2018−2022) (Scheme 1). First, we opted to provide an updated report on CuS‐based PTAs. Most prior studies focused on the synergistic therapies of CuS‐based tumor PTT, such as chemotherapy, radiotherapy, dynamic therapies (photodynamic therapy (PDT), chemodynamic therapy (CDT), and sonodynamic therapy (SDT)), immunotherapy, gene therapy, gas therapy, and magnetic hyperthermia (MHT), owing to a decrease in isolated cases of tumor inhibition using CuS‐based PTT. Here, we further classified these CuS‐based PTAs into two groups: (1) PTT combined with a single treatment; and (2) PTT combined with two or more therapies. The pros and cons of these approaches are outlined in Table 1. Finally, we summarized these therapeutic techniques, outlined the limitations of the current approaches, and suggested potential areas for improvement.

**TABLE 1:** The advantages and disadvantages of copper sulfide (CuS)‐based photothermal therapy (PTT) combined with these therapeutic strategies.

PTT combined with therapies		Advantages	Disadvantages
Chemotherapy		Relieving tumor hypoxia; reversing chemo‐resistance	Adverse effects
Radiotherapy		Relieving tumor hypoxia; reversing radio‐resistance	Adverse effects
Dynamic therapies	PDT	Relieving tumor hypoxia to improve PDT; inhibition of heat shock proteins	Limited light penetration; two different excitation lights needed in PTT/PDT
	CDT	Trigger the release of CDT agents	Insufficient H_2_O_2_; inappropriate pH value
	SDT	Relieving tumor hypoxia to improve SDT; trigger the release of SDT agents	Insufficient therapy effect
Immunotherapy		Increasing the immune response; inhibition of metastasis and recurrence	Low response rate; immune‐related adverse effects
Gene therapy		Inhibition of heat shock proteins	Insufficient gene transport efficiency
Gas therapy		Inhibition of heat shock proteins	Insufficient gas production
MHT		Make up for the deficiency of light penetration in PTT	Low magnetic energy absorption

## 2 CuS‐BASED PTT

Owing to its non‐invasive and practical benefits, PTT with NIR light for tumor therapy has attracted considerable interest. CuS NPs are often employed as PTAs. In general, three goals are proposed for CuS NPs: (1) increase the PCE of CuS NPs; (2) synthesize multi‐modal imaging and therapy‐integrated CuS NPs; (3) construct NIR‐II absorbance therapeutic nanoplatforms that comprise CuS NPs that are enough for deeper tissue penetration. Table S1 provides a summary of these CuS‐based PTT nanoplatforms.

To improve the PCE of CuS NPs, increasing the concentration may enable an increase in the absorbance. However, this strategy only works to a certain extent. Increasing the PCE using CuS NPs in composite nanomaterials is the more practical approach. For example, CuS NPs typically have a PCE in the range of 20%−40%, but when combined with other compounds, the PCE may reach as high as 82.4%.^[^ ^15^ ^]^

**FIGURE 1:**
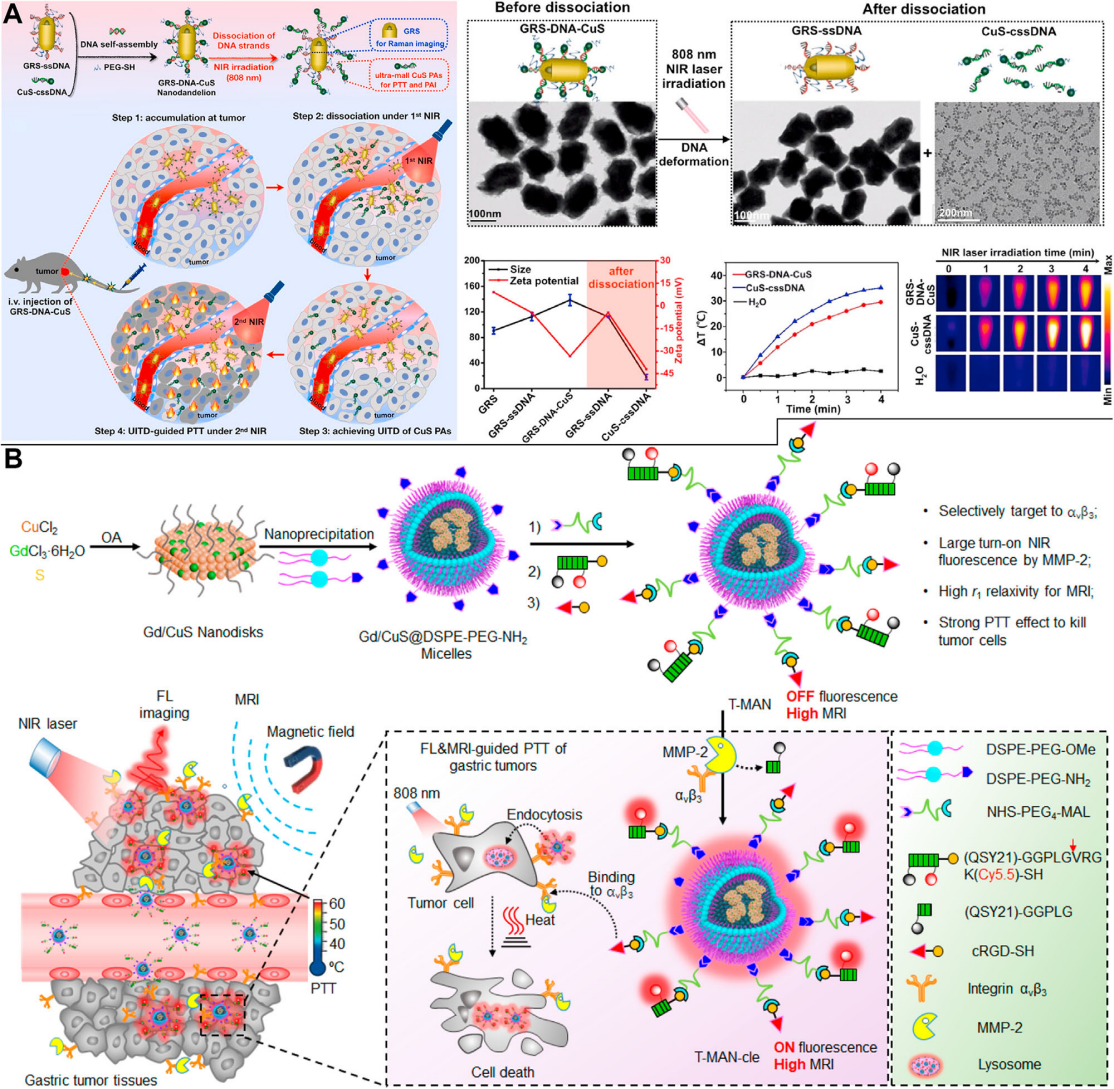
Copper sulfide (CuS)‐based photothermal therapy (PTT). (A) Two rounds of irradiation on a DNA‐assembled “nanodandelion” causing the dissociation of gold nanorods and ultrasmall CuS nanoparticles (NPs) and leading to the elimination of tumors. Reproduced with permission.^[^ ^18^ ^]^ Copyright 2022, Elsevier. (B) The use of a tumor‐targeted and MMP‐2 activatable nanoprobe for MR and fluorescence imaging of gastric tumors and preventing lymph node metastases in PTT. Reproduced with permission.^[^ ^21^ ^]^ Copyright 2019, American Chemical Society.

Irradiation sources for CuS‐based PTT are often lasers in the NIR range (700–900 nm, e.g., 808 nm laser).^[^ ^16^ ^]^ According to Beer–Lambert law, the concentration and extinction coefficient of PTAs significantly influence PCE. However, the accumulation of PTAs in tissues was restricted. Thus, improving the extinction coefficient to contribute to PCE is likely to be more feasible. Accordingly, including more components to improve the PCE is one of the logical courses of action. For example, polydopamine (PDA), a melanin analog, was used to stabilize CuS NPs. Subsequently, the NPs were chelated with iron ions for T_1_‐weighted magnetic resonance (MR) imaging‐guided PTT. With 808 nm laser irradiation, this nanoplatform achieved a PCE of 37.4%, which is approximately three times higher than that of PDA NPs alone. Subsequently, irreversible tumor ablation was performed.^[^ ^17^ ^]^ A DNA‐assembled “nanodandelion” exemplifies an approach designed to enhance the tumor‐killing potential of PTT via repeated exposure to NIR light. Non‐uniform intra‐tumor distribution of PTAs is a critical cause for poor PTT performance. Considering the weaker binding energy of hydrogen bonds than that of covalent bonds, the complementary single‐strand DNA on both the gap‐enhanced rod‐shaped gold structure and ultra‐small CuS NPs were conjugated to create a “nanodandelion.” Notably, the hydrogen bonds were explosively broken within 1 min under the first Raman imaging‐guided NIR light irradiation (808 nm), resulting in the complete dissociation of gold nanorods and ultrasmall CuS NPs. Furthermore, the local concentration of ultrasmall CuS NPs could be rapidly enhanced under minimal tumor temperature rise, leading to uniform intra‐tumor distribution. Subsequently, NIR light irradiation guided by photoacoustic (PA) imaging eradicated 60% of the tumors (Figure 1A).^[^ ^18^ ^]^

PTT must involve laser irradiation when PTAs have accumulated to a substantially high degree in the tumor tissues. Thus, visualization of PTA distribution in vivo is crucial. Using PTAs and multimodal imaging may close this gap and enable for more thorough monitoring of the PTT processes. Such monitoring facilitates the use of CuS NPs as both PTAs in PTT and contrast media in multimodal imaging, as the paramagnetic Cu(II) in CuS NPs can be used as a contrast agent for T_1_‐weighted MR imaging, ^64^Cu radiolabeling can be used for positron emission tomography (PET) imaging, and CuS NPs themselves can be used as contrast agents for PA imaging. To achieve multimodal imaging (MR, PET, and PA imaging) and efficient clearance through the renal‐urinary system, Zhou et al. employed bovine serum albumin (BSA) to dope Mn^2+^ and ^68^Ga^3+^ into CuS NPs without chelating agents. Notably, the inhibition of ovarian tumor development by PTT with a 980 nm laser was statistically significant.^[^ ^19^ ^]^ Regarding lymph node metastasis, Shi et al. produced a wide range of CuS‐based NPs for use in multimodal imaging‐guided PTT. These researchers created Arg‐Gly‐Asp polypeptide (RGD)‐CuS‐Cy5.5 nanoprobes that could enter MNK45 gastric carcinoma cells through RGD‐α_v_β_3_ receptor‐mediated endocytosis and then drain into sentinel lymph nodes. Local PTT led to the complete death of metastatic tumor cells in the SLN, and CT and 808 nm NIR laser‐excited fluorescence imaging permitted a clear distinction of SLN metastases from stomach cancer.^[^ ^20^ ^]^ Shi et al. also developed a tumor‐targeted and matrix metalloproteinase (MMP)‐2 activatable nanoprobe for high spatial resolution MR and low background fluorescence imaging of gastric tumors and lymph node metastases, in which Gd‐doped CuS NPs were modified with cyclic RGD and MMP‐2 cleavable fluorescent substrates. These probes were employed in 808‐nm tumor PTT, which led to an improved PCE of up to 70.1% (Figure 1B).^[^ ^21^ ^]^

**FIGURE 2:**
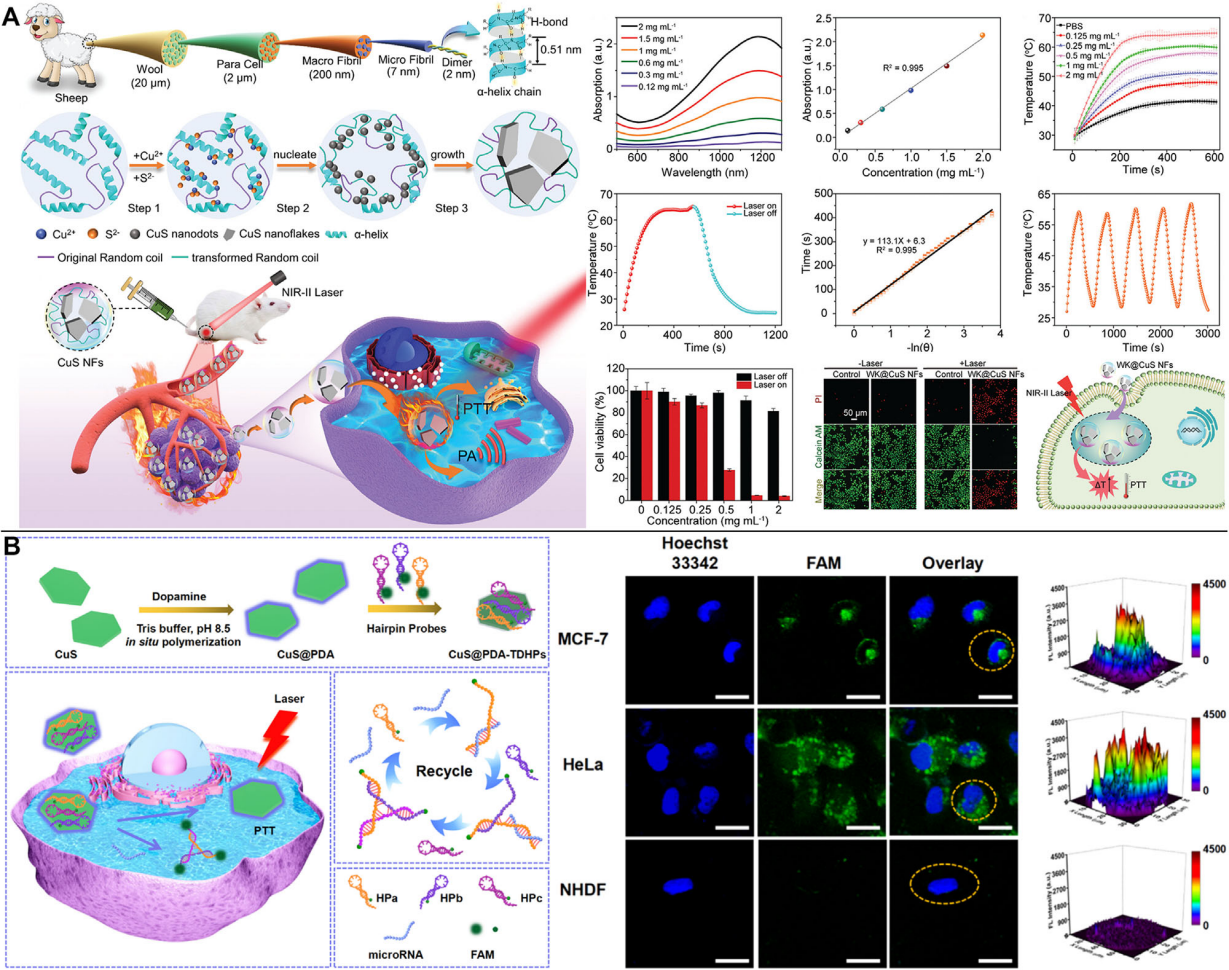
Copper sulfide (CuS)‐based photothermal therapy (PTT). (A) The 1064‐nm laser PTT and PA imaging owing to the controlled design and manufacturing of 2D CuS nanoflakes through the α‐helix to random coil transition in keratin. Reproduced with permission.^[^ ^25^ ^]^ Copyright 2022, The Royal Society of Chemistry. (B) By combining fluorescence imaging and 1064‐nm laser PTT, cancer cells distinguished from normal cells and killed using polydopamine (PDA)‐functionalized CuS nanosheets with three DNA hairpin probes. Reproduced with permission.^[^ ^29^ ^]^ Copyright 2022, American Chemical Society.

As the NIR‐I window has limitations in terms of penetration depth and maximum permissible exposure power densities, the NIR‐II region (1000–1700 nm) and especially the NIR‐IIa region (1300–1400 nm) are considered more promising for tumor diagnosis and treatment (e.g., 808 nm, 0.33 W cm^−2^; 980 nm, 0.726 W cm^−2^; 1064 nm, 1.0 W cm^−2^).^[^ ^22^, ^23^ ^]^ Research from the emergence of the field highlighted the 808‐nm laser as the most popular choice for CuS‐based PTT owing to the earlier and inexpensive development of laser devices that operate in the NIR‐I region. CuS NPs might better fit with the wavelength range in the NIR‐II region as their absorbance increases steeply, beginning at approximately 900 nm. CuS NPs could efficiently eradicate MCF‐7 cells when exposed to a 1064‐nm NIR laser. Further, CuS NPs successfully transformed the environmental carcinogen, p‐nitrophenol, into the drug‐significant p‐aminophenol.^[^ ^24^ ^]^ Tumors might be treated with 1064‐nm laser PTT and monitored with PA imaging owing to the controlled design and precise manufacturing of 2D CuS nanoflakes through the keratin α‐helix to random coil transition (Figure 2A).^[^ ^25^ ^]^ Enhanced absorbance in both the NIR‐I and NIR‐II windows was achieved using the novel Prussian blue@poly‐acrylic acid/CuS Janus NPs. The 1064‐nm laser irradiation resulted in enhanced tissue penetration, demonstrating the ability of the NPs to treat malignancies in deep tissues.^[^ ^26^ ^]^ Furthermore, (3‐carboxypropyl)triphenylphosphonium bromide was used to modify CuS NPs, which were subsequently encapsulated with hyaluronic acid (HA) to target CD44. Tumor cells were eliminated in PTT in vitro and in vivo when CuS NPs accumulated at concentrations of almost 90% in the mitochondria.^[^ ^27^ ^]^ PTT with a 1275‐nm laser was technically possible in addition to PPT with a 1064‐nm laser. Better in vitro cell ablation and in vivo deep tissue anticancer capability were obtained using the 1064‐nm laser than that using the 808‐nm laser.^[^ ^28^ ^]^

Multimodal imaging techniques were used to guide and monitor PTT. By combining precise fluorescent imaging‐guided PTT with endogenous cancer miRNA detection, Yang et al. have created a novel theranostic nanoplatform. The presence of tumor‐specific miRNA‐155 enabled the ternary complex DNA hairpin anchored on CuS@PDA nanosheets to form a three‐arm branching junction structure, leading to fluorescence recovery and effective discrimination of cancer and normal cells. In vivo studies using laser PTT at 1064 nm revealed strong anticancer therapeutic efficacy (Figure 2B).^[^ ^29^ ^]^ Researchers used a nanogel that included Gd and CuS for MR/PA imaging to treat cancers that overexpress folate receptors. This facilitated precise tumor localization and guided targeted PTT under 1064‐nm laser irradiation.^[^ ^30^ ^]^ Incorporating ^99m^Tc‐M‐CuS‐polyethylene glycol (PEG) into the cell membrane enabled single photon emission computed tomography/PA imaging‐guided tumor PTT. The increased therapeutic efficacy of 1064‐nm laser PTT was demonstrated when labeled ^99m^Tc was used to increase cell uptake to M‐CuS‐PEG by causing cell cycle G2/M arrest.^[^ ^31^ ^]^

To achieve these three goals, several CuS‐based PTAs have been created. In particular, noble metal‐based PTAs whose LSPR may further activate resonance energy transfer (RET) to CuS NPs were integrated with CuS NPs to enhance the PCE. Besides the PA imaging capability of CuS NPs, the contrast agents of MR, PET, and CT imaging were integrated to contribute to multimodal imaging and therapy. Such integration would provide more information, such as therapeutic process, chemotherapeutic drug release, and physiological microenvironment monitoring (e.g., tumor hypoxia). CuS NP absorbance indicates that the NIR‐II region is better for CuS NP‐based PTT owing to its deeper tissue penetration; however, only a few studies have concentrated on this area. Although tumor suppression has been accomplished with CuS‐based PTT, the fundamental issue with single PTT is its restricted therapeutic effectiveness. CuS‐based PTT combined with complementary therapies must be used to achieve the best possible results from PTT.

## 3 CuS‐BASED PTT COMBINED WITH ONE THERAPY

### 3.1 Chemotherapy

PTT, in addition to chemotherapy, has several benefits. For instance, PTT may improve blood flow, aid in the penetration and diffusion of chemotherapeutic drugs into tumor tissues, and prevent tumor metastasis and recurrence, even during the administration of chemotherapy. Several strategies are available for loading chemotherapy drugs onto nanoplatforms, including drug adsorption, drug loading into hollow mesoporous CuS NPs, or co‐loading CuS NPs with chemotherapeutic agents in polymers or on other nanocarriers. Table 2 and Table S2 summarize these nanoplatforms for CuS‐based PTT combined with chemotherapy.

**TABLE 2:** Copper sulfide (CuS)‐based photothermal therapy (PTT) combined with chemotherapy or radiotherapy.

Names of CuS‐based nanoplatforms	Target ligands	Therapies	Tumor cell lines	Drugs	DEE (%)	DLC (%)	PCE (%)	Lasers used in vitro	Lasers used in vivo	Ref.
CTR/DOX	RGD	Chemotherapy	4T1	DOX	50	8.7	/	808 nm, 980 nm, 2.0 W cm^−2^, 3 min	980 nm, 0.726 W cm^−2^, 5 min	[34]
SNT‐LA‐CuS‐PEG/DOX	LA	Chemotherapy	HepG2	DOX	/	12	/	808 nm; 2.0 W cm^−2^; 5 min	808 nm; 2.0 W cm^−2^; 5+5 min	[35]
SPDG	SA	Chemotherapy	HepG2	DOX	77.4	3.54	/	980 nm, 2.0 W cm^−2^, 5 min	980 nm, 2.0 W cm^−2^, 6 min	[36]
CuSCDB@MMT7	T7	Chemotherapy	MCF‐7, 4T1, THp‐1, RAW 264.7	Bortezomib	81.9	20.5	39.7	808 nm, 0.5 W cm^−2^, 5 min	808 nm, 0.3 W cm^−2^, 5 min	[37]
IDHCuSNP@B16F10	Cell membrane	Chemotherapy	B16F10	DOX	ICG: 98, DOX: 85	/	/	808 nm, 1.0 W cm^−2^, 5 min	808 nm, 0.5 W cm^−2^, 5 min	[38]
CuS‐SF@CMV NPs	Cell membrane	Chemotherapy	HepG2	Sorafenib	88	/	34.3	808 nm, 1.0 W cm^−2^, 5 min	808 nm, 0.6 W cm^−2^, 5 min	[39]
CuS DENPs	RGD	Chemotherapy	4T1	DOX	/	/	57.8	1064 nm, 0.6 W cm^−2^, 5 min	1064 nm, 0.6 W cm^−2^, 5 min	[43]
CuS@NGs‐LA/DOX	LA	Chemotherapy	HepG2	DOX	78.4	/	31.1	1064 nm, 0.6 W cm^−2^, 10 min	1064 nm, 0.6 W cm^−2^, 10 min	[47]
FaPCH NPs	FA	Chemotherapy	HeLa, A549	DOX	/	/	19	808 nm, 1.0 W cm^−2^, 10 min	808 nm, 1.0 W cm^−2^, 5 min	[48]
CMDMm	Cell membrane	Chemotherapy	4T1, HepG2	DOX	/	/	/	980 nm, 1.0 W cm^−2^, 6 min	980 nm, 1.0 W cm^−2^, 6 min	[49]
FA‐BSA/CuS@ZIF‐8‐QT	FA	Chemotherapy	B16F10	Quercetin	/	24	/	808 nm, 2.0 W cm^−2^, 5 min	808 nm, 1.0 W cm^−2^, 5 min	[54]
CuS‐ATMi@TGF‐β NPs	TGF‐β	Chemotherapy	HepG2	ATM inhibitor	93.5	86.6	32.6	808 nm, 0.5 W cm^−2^, 5 min	808 nm, 0.3 W cm^−2^, 5 min	[55]
(UCNPs/CuS)@MnO_2_	/	Radiotherapy	HepG2, CT26	/	/	/	/	1064 nm, 0.5 W cm^−2^, 8 min	1064 nm, 0.5 W cm^−2^, 10 min	[64]
CuS@CeO_2_	/	Radiotherapy	HepG2	/	/	/	/	1064 nm, 0.5 W cm^−2^, 10 min	1064 nm, 0.5 W cm^−2^, 10 min	[65]

Abbreviations: ATM, ataxia‐telangiectasia mutated; DEE, drug encapsulation efficiency; DLC, drug loading content; DOX, doxorubicin; PCE, photothermal conversion efficiency.

Numerous case studies involving the use of CuS‐based PTT combined with chemotherapy have been presented, highlighting the effectiveness of this approach for tumor treatments. For instance, the use of doxorubicin (DOX)‐encapsulated 3D CuS hollow nanoflowers had an enhanced tumor suppression rate than that of PTT or chemotherapy alone.^[^ ^32^ ^]^ The hydrophilic 5‐fluorouracil and hydrophobic paclitaxel (PTX) were successfully encapsulated in CuS‐ZnS using electrostatic interactions and a chitosan shell. Finally, PTT combined with dual‐drug chemotherapy significantly reduced tumor growth.^[^ ^33^ ^]^

**FIGURE 3:**
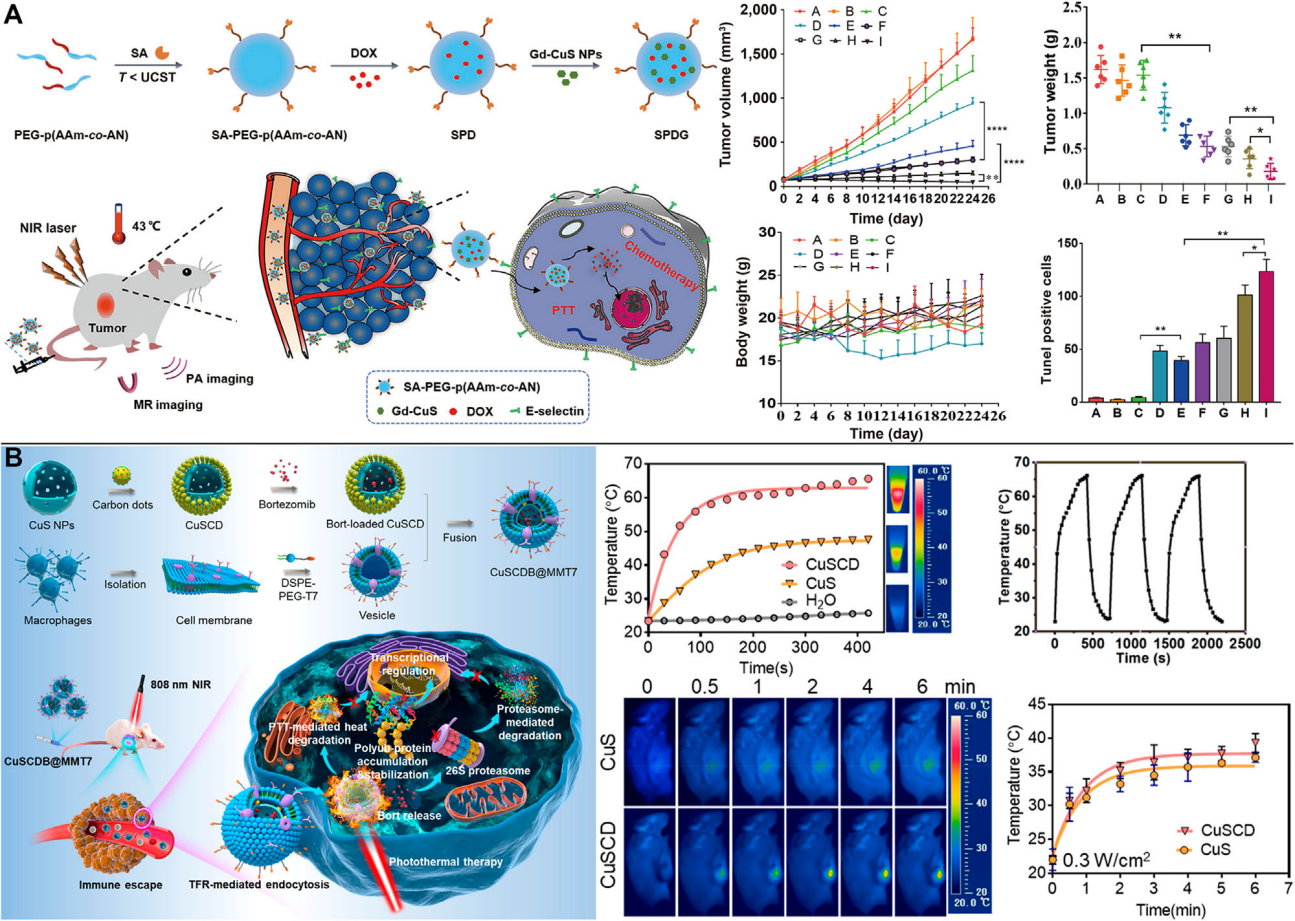
Copper sulfide (CuS)‐based photothermal therapy (PTT) combined with chemotherapy. (A) Specifically targeting and successfully inhibiting hepatocellular carcinoma cells in synergistic chemo/PTT with MR/PA dual‐modality imaging. Reproduced with permission.^[^ ^36^ ^]^ Copyright 2021, Springer Nature. (B) CuSCDB@MMT7, encapsulating the bortezomib, enabling PTT combined with chemotherapy. Reproduced with permission.^[^ ^37^ ^]^ Copyright 2020, American Chemical Society.

The accumulation of drugs in tumor tissues may be improved by altering the targeting ligands on the surface of drug delivery nanocarriers. One standard method of delivering drugs into tumor cells involves coupling the drugs to specific ligands and actively targeting the cells. For instance, the synergistic chemo/PTT was at least 3.53‐fold more effective than any single therapy and effectively suppressed tumor liver metastasis after RGD was coupled to CuS NPs, which demonstrated an actively targeting action and enhanced DOX uptake into tumor cells.^[^ ^34^ ^]^ When combined with targeted chemotherapy, the PTT, which comprised DOX‐loaded hollow porous silica nanotubes dotted with CuS NPs and conjugated with lactic acid moieties for tumor targeting, could achieve improved tumor suppression.^[^ ^35^ ^]^ Using MR/PA dual modality imaging‐guided synergistic chemo/PTT, polymer micelles loaded with DOX/Gd‐CuS NPs actively targeted hepatocellular carcinoma cells via the affinity of overexpressed E‐selectin, leading to substantial tumor suppressive effect in vivo (Figure 3A).^[^ ^36^ ^]^

Using cell membranes, such as those of macrophages or tumor cells, to encase NPs is a novel tumor‐targeting approach comparable to ligand targeting. Characterized hollow‐structured CuS NPs composited with carbon dots (CuSCDs) could efficiently infiltrate 4T1 tumor cells and promote favorable immune evasion after coating with the macrophage membrane hybridized with T7 peptide. To reduce the risk of severe toxic side effect and poor blood stability of bortezomib, CuSCDs could be used and involved in heat‐stability of cell proliferation and survival associated with various substrates through the ubiquitin‐dependent proteasomal degradation pathway (Figure 3B).^[^ ^37^ ^]^ The accumulation of NPs in vitro and in vivo models of B16F10 cells facilitated the chemo/PTT to achieve high tumor ablation rates. This achievement was attributed to the encapsulation of DOX and indocyanine green (ICG)‐loaded hollow CuS NPs in the melanoma cell membrane.^[^ ^38^ ^]^ Alternatively, sorafenib, anti‐VEGFR antibodies, and hollow CuS NPs could be encased in tumor cell/macrophage hybrid membranes, improving the targeting capability and internalization in tumor cells, and leading to synergistic chemo/PTT. Accordingly, a tumor inhibitory effect was achieved with sorafenib and anti‐VEGFR antibodies. These two treatments blocked the Ras/Raf/MEK/ERK and PI3K/AKT pathways, respectively, which were involved in cell proliferation and angiogenesis in malignancies.^[^ ^39^ ^]^

The chemotherapeutic effect may be improved using nanocarriers internalized in cells and activated to release their cargo under certain conditions. RET between a variety of gold cores and CuS shells (Au‐CuS YSNPs) was evaluated by Chang et al. Matching the wavelength of incident light to the LSPR absorption wavelength of gold cores activates RET from gold cores to CuS shells, markedly increasing the magnitude of electron‐hole pairs in the CuS shells and promoting the d‐d energy band transition of Cu(II) ions owing to the excited electron. Owing to this phenomenon, the PTT and PDT of CuS‐based NPs shall be markedly improved. DOX was loaded into the gap between the gold core and CuS shells, and the Au‐CuS YSNPs were then coated with a thermo‐responsive polymer to regulate drug release based on temperature. Tumor growth was successfully suppressed owing to the synergistic therapeutic action of PTT with DOX‐loaded nanocarriers under 980‐nm laser irradiation.^[^ ^40^ ^]^ Notably, CuS nanogels could load significant amounts of DOX. The large‐size framework of this is hybrid nanogel self‐destructed at a high temperature, enabling for the rapid release of small NPs into the tumor to release DOX at a 26.3‐fold release rate, as demonstrated by PA imaging, thereby significantly inhibiting tumor progression in vivo.^[^ ^41^ ^]^

**FIGURE 4:**
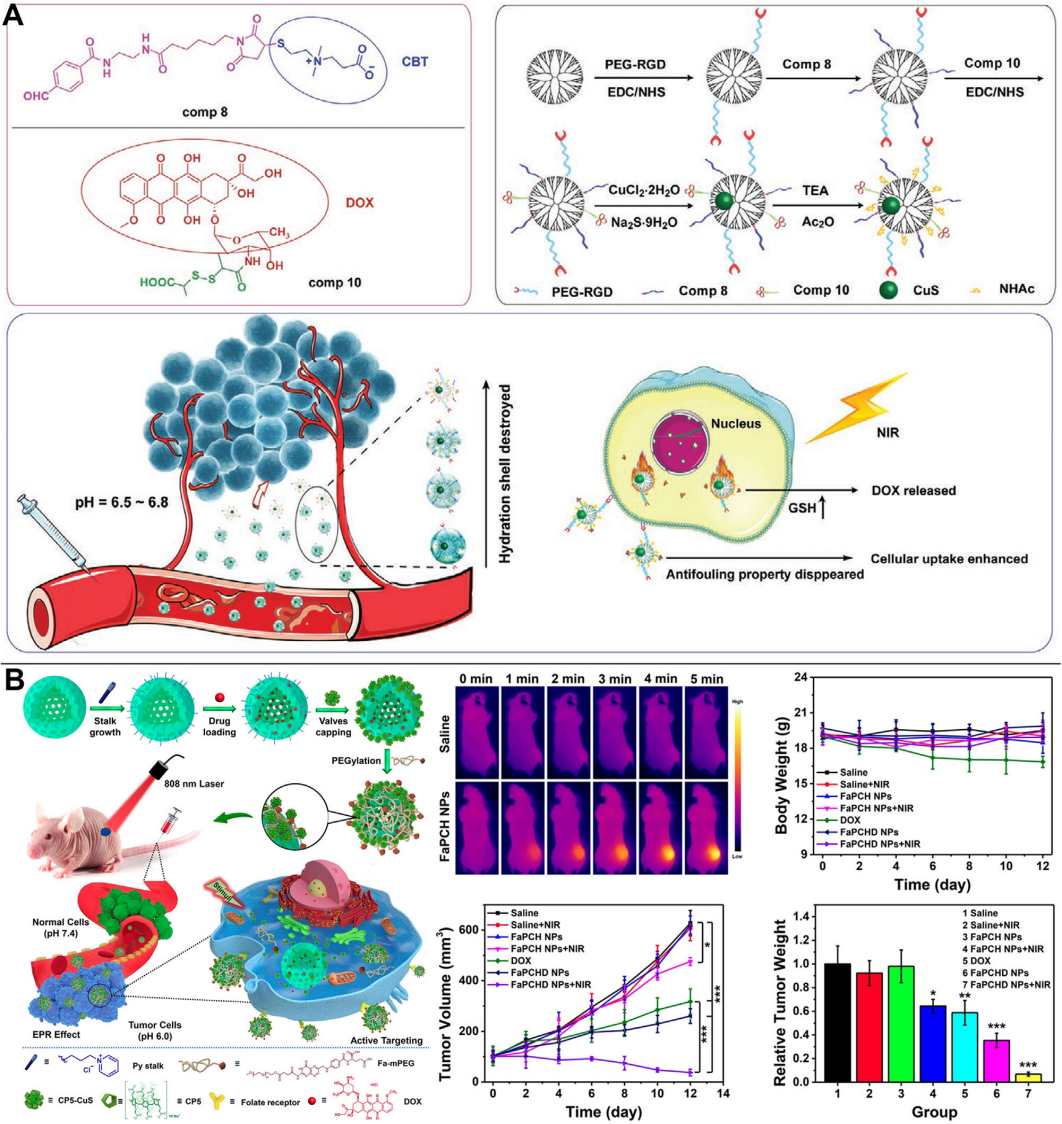
Copper sulfide (CuS)‐based photothermal therapy (PTT) combined with chemotherapy. (A) CuS grew in situ inside the dendrimer enabling synergistic chemo/PTT, whereas the pH‐responsive material eliminating the hydrated layer in a tumor microenvironment with a pH of 6.5–6.8. Reproduced with permission.^[^ ^43^ ^]^ Copyright 2021, John Wiley & Sons. (B) Temperature, pH, competitive binding, and near‐infrared (NIR) light stimulation activating the drug release from pyridine‐modified HMSN‐loading doxorubicin (DOX) during synergistic chemo/PTT. Reproduced with permission.^[^ ^48^ ^]^ Copyright 2020, Ivyspring International Publisher.

Using a co‐precipitation/assembly method, Fe‐metal organic framework (Fe‐MOF) shells were formed on the surface of CuS nanoplates to create MOF nanoplatforms. The high PCE (39.7%) of CuS@Fe‐MOF NPs could enable DOX loading followed by its release in an acidic environment. Adjuvant chemo/PTT synergistic tumor treatment might be aided by MR imaging.^[^ ^42^ ^]^ Additionally, thiolated *N,N*‐dimethyl‐cysteamine‐carboxybetaine played a pH‐responsive role and could induce charge reversal to promote tumor uptake/penetration in the tumor microenvironment at pH 6.5–6.8. Glutathione (GSH) could also trigger and induce DOX release in the redox environment.^[^ ^43^ ^]^ This work involved CuS growing in situ inside dendrimers coupled with active targeting of the ligand, RGD, for DOX delivery (Figure 4A). The disulfide bonds between CuS and nanocarriers could be disrupted by GSH in the tumor redox environment, enabling DOX release and biodegradation of the nanocarriers. Enhanced tumor suppression was achieved owing to the high PCE (51.5%) that enabled PTT after DOX delivery by nanocarriers to the tumor regions.^[^ ^44^ ^]^ In addition, Niu et al. created a chemo/PTT synergistic nanoplatform composed of CuS NPs and hollow mesoporous silica NPs (HMSNs), with the HMSNs serving as payloads for DOX and thioglycolic acid‐modified chitosan serving as a bridge to covalently connect the HMSNs and CuS NPs. The synergistic chemo/PTT increased post‐treatment survival in tumor‐bearing mice from 34 to 58 days. CuS NPs had a gatekeeper function to induce DOX release only in the tumor GSH‐rich microenvironment.^[^ ^45^ ^]^ Similarly, Cheng et al. employed CuS NPs as gatekeepers in a yolk–shell structured periodic mesoporous organosilica NPs, with DOX loaded in space within them with a high drug loading capacity of 386 mg g^−1^ and released in response to an intracellular acidic/redox environment and external NIR stimulation.^[^ ^46^ ^]^

When CuS NPs were encapsulated in cationic poly(N‐vinylcaprolactam), the same internal acidic/redox environment and external NIR could trigger DOX release, enabling effective chemo/PTT. This nanoplatform was discovered to significantly limit tumor recurrence and eradicate advanced tumors in mice (large volume 0.15−0.20 cm^3^).^[^ ^47^ ^]^ To actively target tumors, pyridine‐modified HMSNs containing DOX were capped with CuS NPs through supramolecular host–guest interactions and folic acid (FA) was added via PEGylation. These nanoplatforms can be activated by a combination of four stimuli (temperature, pH, competitive binding, and NIR light) to release drugs in a regulated manner during synergistic chemo/PTT (Figure 4B).^[^ ^48^ ^]^ Additionally, CuS@MSN was employed as PTAs and nanocarriers to load DOX by Jia et al., which led to the development of “dual‐lock” NPs named CuS@MSN/DOX@MnO_2_@membrane. To release DOX from MnO_2_ and CuS@MSN, both GSH and a slightly acidic microenvironment were used. MnO_2_ reduction and degradation in the tumor microenvironment could provide T_1_‐weighted MR imaging‐relevant Mn^2+^.^[^ ^49^ ^]^ DOX was loaded into CuS nanocages with a mesoporous and hollow structure using a similar technique. MnO_2_ nanoshells regulated the release of DOX, and MnO_2_ was triggered to produce Mn^2+^ for MR imaging and fluorescent imaging using DOX for tumor PTT.^[^ ^50^ ^]^

**FIGURE 5:**
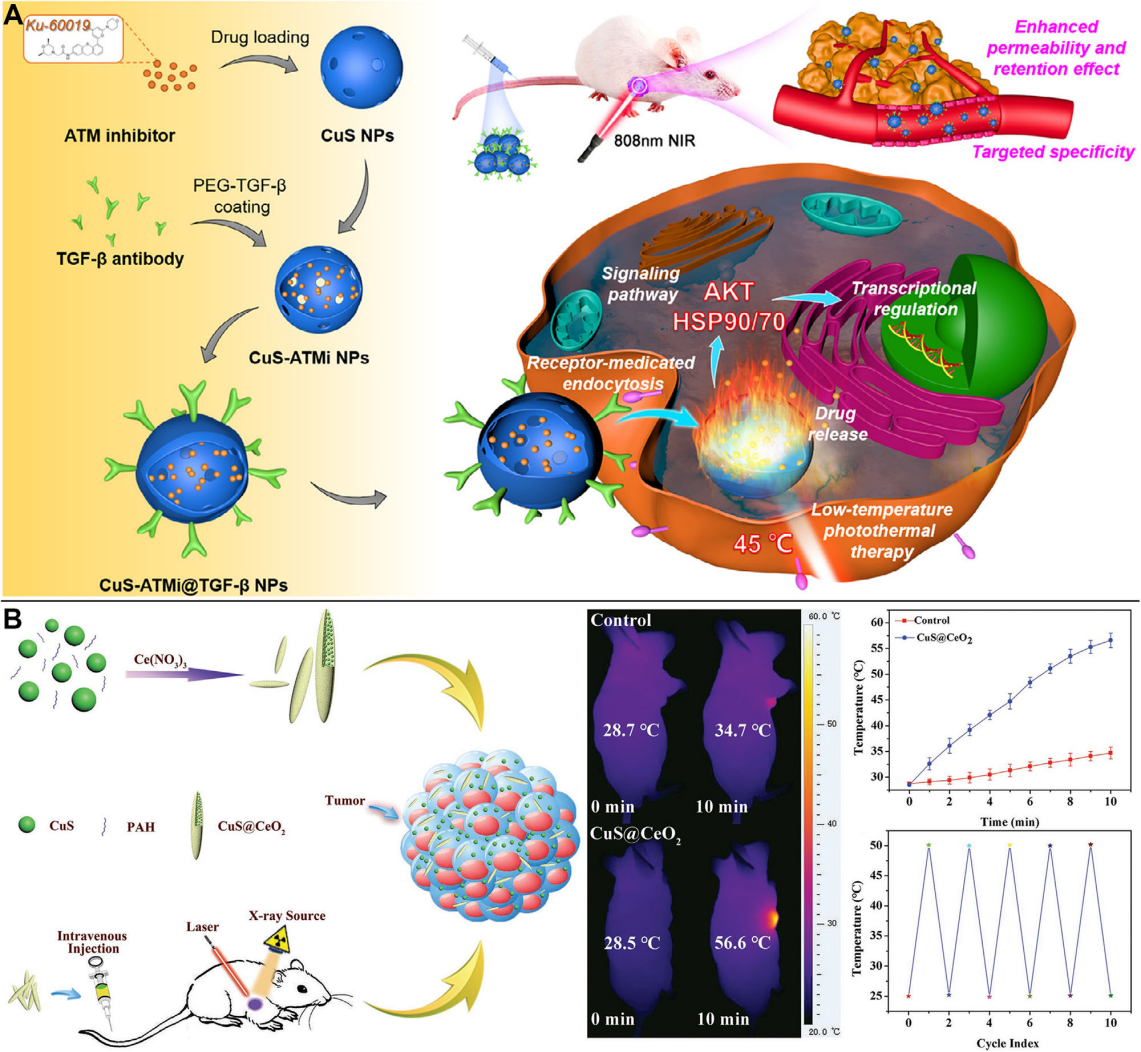
Copper sulfide (CuS)‐based photothermal therapy (PTT) combined with chemotherapy or radiotherapy. (A) CuS‐ATMi@TGF‐β nanoparticles (NPs) loaded with ataxia‐telangiectasia mutated (ATM) inhibitors suppressing hepatocellular carcinoma cell growth in hypothermic PTT and ATM treatment via downregulation of heat shock proteins. Reproduced with permission.^[^ ^55^ ^]^ Copyright 2021, Elsevier. (B) Spindle‐shaped CuS@CeO_2_ relieving tumor hypoxia, decreasing the necessary radiation dosage, and preventing tumor recurrence through catalyzing the conversion of endogenous H_2_O_2_ to O_2_ during PTT. Reproduced with permission.^[^ ^65^ ^]^ Copyright 2020, John Wiley & Sons.

Although the model anticancer agent, DOX, was utilized in the aforementioned chemotherapeutic combinations, other agents may also lead to a synergistic effect. In particular, for tumor targeting, the combination of Fe(III)‐MOF‐Pt/PEG‐CuS and cisplatin(II) enabled improved synergistic chemo/PTT without causing liver toxicity, which occurs with cisplatin(II) therapy alone.^[^ ^51^ ^]^ The in vivo degradation of disulfiram to diethyldithiocarbamate (DDTC) resulted in the formation of the complex Cu(DDTC)_2_, which killed tumor cells. Consequently, a unique multifunctional CuS‐DDTC nanoplatform permitted the synergistic effect involving PTT and DDTC chemotherapy.^[^ ^52^ ^]^ CuS NPs were synthesized in situ using antitumor copper diethyldithiocarbamate [Cu(DTC)_2_], with high PCE increasing the cytotoxicity of PTT and efficiently abating B16 melanoma tumors with the assistance of PA imaging‐guided ablation.^[^ ^53^ ^]^ Quercetin has poor bioavailability and water solubility and is unstable in an alkaline environment. However, these drawbacks may be mitigated using synergistic chemo/PTT, including zeolitic imidazolate framework‐8 (ZIF‐8) with quercetin and CuS NPs as a chemotherapeutic agent and the PTAs, respectively. When FA‐BSA was used to achieve dynamically targeted drug delivery, drug accumulation was increased in tumor tissues, and synergistic treatment markedly enhanced the anticancer effect.^[^ ^54^ ^]^ CuS‐ATMi@TGF‐NPs, loaded with ataxia‐telangiectasia mutated (ATM) inhibitors, could actively target tumors with an anti‐TGF antibody and substantially reduce tumor development. The combination of hypothermic PTT and ATM treatment exhibited a synergistic effect in preventing the proliferation of hepatocellular carcinoma cells by lowering heat shock protein levels (Figure 5A).^[^ ^55^ ^]^ Feng et al. used intracellular bottom‐up synthesis of CuS NPs to create these NPs, ultimately leveraging the elevated content of endogenous H_2_S in tumor cells. Cu‐meloxicam complexes were loaded with the assistance of human serum albumin. Ultrasmall CuS NPs were created for efficient tumor PTT and meloxicam release and acted by reacting competitively with endogenous H_2_S. However, the anti‐inflammatory properties of meloxicam have been demonstrated to provide an additional anticancer benefit and reduce tumor recurrence and metastasis.^[^ ^56^ ^]^ PTT‐induced inflammation may be reduced using a new nanocarrier called BiOI@CuS NPs co‐loading DOX, aspirin, phenacetin, and caffeine.^[^ ^57^ ^]^

Some of the major drawbacks of the CuS‐based synergistic chemo/PTT are listed: (1) The synergistic effect of other chemotherapeutic drugs besides DOX should be further explored and evaluated. (2) Many studies on synergetic treatment have identified a combination index (CI) that can be calculated using the following equation:

$$CI\;=\frac{C_{1}}{C_{m1}}\;+\frac{C_{2}}{C_{m2}}$$

where *C_1_* is the concentration of PTAs under constant NIR irradiation and *C_2_* is the concentration of drugs in the same synergetic therapeutic system. *C_m1_* and *C_m2_* indicate the concentrations of PTAs or drugs employed in a single therapy, causing the same therapeutic effect.^[^ ^58^, ^59^, ^60^ ^]^ This CI was initially employed in dual‐drug synergetic therapy. However, laser powers and irradiation time were not fully considered when utilized in synergetic chemo/PTT; therefore, a more appropriate calculated approach should be recommended.

### 3.2 Radiotherapy

One well‐known cause of radio‐resistance in tumor treatment is hypoxia. Radiation‐induced reactive oxygen species (ROS) causes DNA damage and further cellular apoptosis and necrosis. Tumor hypoxia reverses DNA damage in the tumor redox environment and causes oxidative stress in radiotherapy and can lead to the overexpression of HIF‐1α, triggering metabolic reactions that weaken the radiation effect.^[^ ^61^, ^62^, ^63^ ^]^ However, MnO_2_ can create extra H_2_O_2_ at the tumor site to compensate for the O_2_ shortfall in the hypoxic tumor microenvironment, markedly enhancing the therapeutic benefit of radiotherapy. Boosting vascular blood flow using CuS‐based PTT may synergistically improve the effectiveness of radiation. In multimodal upconversion fluorescence, CT, and MR imaging, upconverting NPs (UCNPs) were employed.^[^ ^64^ ^]^ Similar to this instance, researchers produced spindle‐shaped CuS@CeO_2_ to promote tumor tissue penetration and internalization. CeO_2_ acted as a nanoenzyme to accelerate the conversion of endogenous H_2_O_2_ to O_2_ in tumor tissues, enhancing the hypoxic tumor microenvironment by following a similar principle. Notably, the radiation oncology dosages could be lowered, and tumor recurrence was avoided using CuS‐based PTT (Figure 5B).^[^ ^65^ ^]^ Table 2 provides a summary of the nanoplatforms employed in CuS‐based PTT in combination with radiotherapy.

### 3.3 Dynamic therapies

PDT, CDT, and SDT are types of dynamic therapies. Table 3 and Table S3 provide a summary of the nanoplatforms employed in CuS‐based PTT in combination with dynamic therapies.

**TABLE 3:** Copper sulfide (CuS)‐based photothermal therapy (PTT) combined with dynamic therapies.

Names of CuS‐based nanoplatforms	Target ligands	Therapies	Tumor cell lines	Drugs	DEE (%)	DLC (%)	PCE (%)	Lasers used in vitro	Lasers used in vivo	Ref.
BP‐CuS‐FA	FA	PDT	4T1	/	/	/	62.6	808 nm, 1.0 W cm^−2^, 8 min	808 nm, 1.0 W cm^−2^, 8 min	[71]
PCN‐CuS‐FA‐ICG	FA	PDT	MDA‐MB‐231	ICG	/	10.2	/	650 nm, 50 mW cm^−2^, 5 min; 808 nm, 1.0 W cm^−2^, 5 min	650 nm, 50 mW cm^−2^, 10 min; 808 nm, 1.0 W cm^−2^, 10 min	[72]
CUSCs‐PEG‐FA	FA	PDT	HeLa, U14	/	/	/	27.4	808 nm, 1.0 W cm^−2^, 10 min	808 nm, 1.0 W cm^−2^, 10 min	[75]
Ce6‐CuS/MSN@PDA@MnO_2_‐FA NPs	FA	PDT	4T1	Ce6	96.3	11	/	660 nm, 50 mW cm^−2^, 10 min; 808 nm, 2.0 W cm^−2^, 10 min	660 nm, 50 mW cm^−2^, 10 min; 808 nm, 2.0 W cm^−2^, 10 min	[77]
MCIH	HA	PDT	U14, HeLa	ICG	80.22	/	24.9	808 nm, 0.76 W cm^−2^, 10 min	808 nm, 0.76 W cm^−2^, 10 min	[78]
BCGCR	cRGD	CDT	U87mg	/	/	/	30.3	980 nm, 0.8 W cm^−2^, 5 min	980 nm, 0.8 W cm^−2^, 10 min	[82]
CuS‐PGH NMs	HA	CDT	4T1	GOx	/	/	/	1064 nm, 1.0 W cm^−2^, 5 min	1064 nm, 1.0 W cm^−2^, 5 min	[84]
AIBA@CuS‐FA	FA	CDT	KB	AIBA	13.3	11.8	47.5	808 or 1064 nm; 0.5 W cm^−2^; 5 min	808 or 1064 nm; 0.5 W cm^−2^; 10 min	[85]
CuS/HAS‐TAPP	/	SDT	MCF‐7	TAPP	/	27.3	/	808 nm, 2.0 W cm^−2^, 5 min	808 nm, 2.0 W cm^−2^, 5 min	[87]
Pt‐CuS‐P‐TAPP	/	SDT	CT26	TAPP	/	18.5	34.5	808 nm, 0.8 W cm^−2^, 7 min	808 nm, 1.0 W cm^−2^, 7 min	[88]

#### PDT

As CuS NPs can produce Cu^+^ for these light‐driven reactions (${\mathrm{Cu}}^{+}+O_{2}\to {\mathrm{Cu}}^{2+}+\cdot O_{2}^{-}$; $2H^{+}+2\cdot O_{2}^{-}\to H_{2}O_{2}+O_{2}$; ${\mathrm{Cu}}^{+}+H_{2}O_{2}\to {\mathrm{Cu}}^{2+}+\cdot \mathrm{OH}+{\mathrm{OH}}^{-}$), they have been labeled as PDT by some researchers.^[^ ^66^ ^]^ CuS NPs have also been categorized as CDT in the literature because of the •OH generated in a Fenton‐like reaction.^[^ ^67^ ^]^ Our summary follows the classification used in the literature. In addition to PTT, PDT is a popular dynamic therapy choice. To accomplish T_1_‐weighted MR imaging and PDT killing of tumor cells, specific CuS NPs could have their $S_{2}^{-}$‐surface (102) readily oxidized, resulting in the release of Cu^+^ and the production of ROS.^[^ ^68^ ^]^ In addition, PTT/PDT could be used to cause cell apoptosis by either using the metal‐free covalent organic framework (COF) or directly generating CuS NPs activated by endogenous H_2_S in vivo.^[^ ^69^ ^]^

**FIGURE 6:**
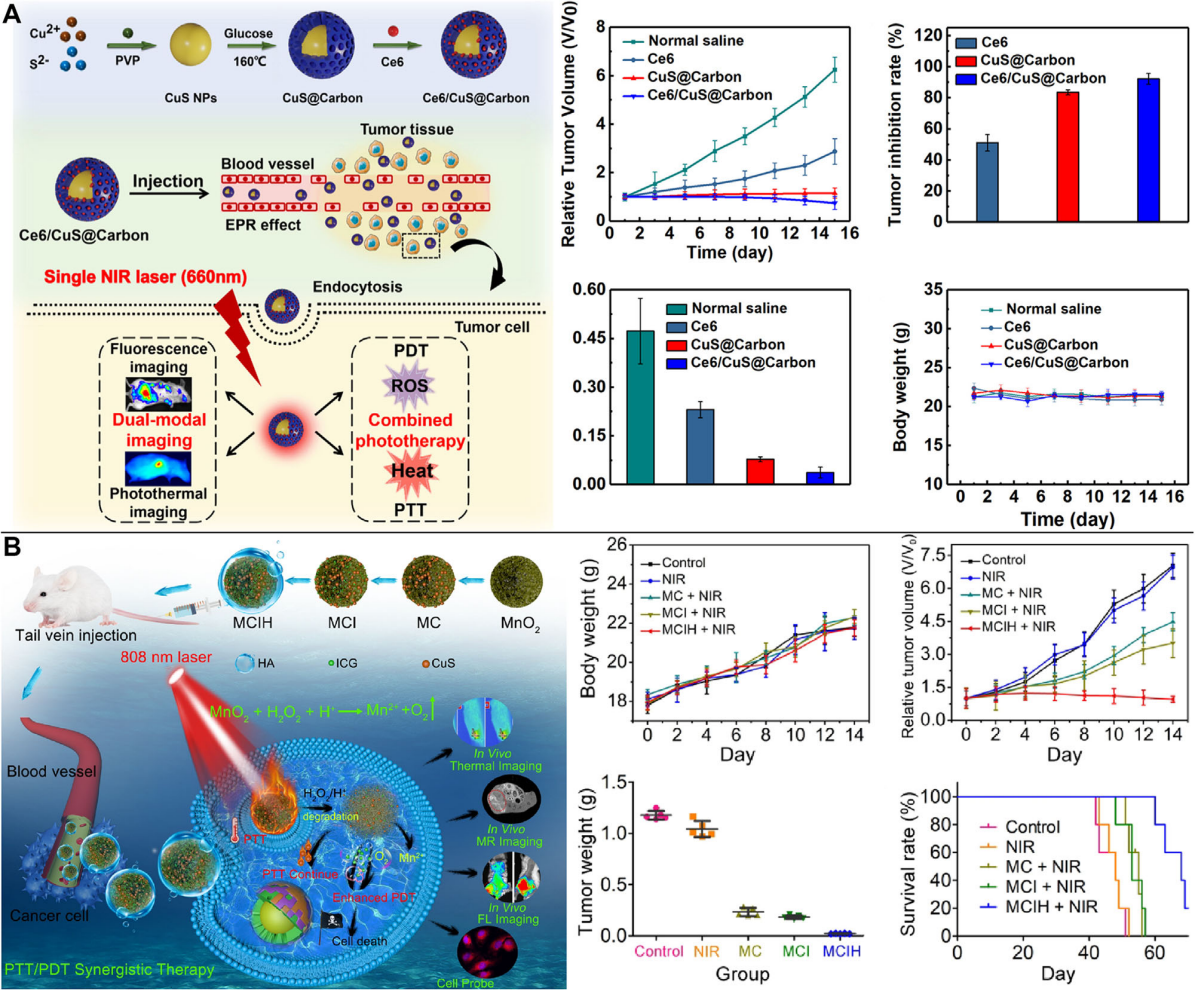
Copper sulfide (CuS)‐based photothermal therapy (PTT) combined with photodynamic therapy (PDT). (A) The use of CuS nanoparticles (NPs) and Ce6 in CuS@Carbon enabling PTT/PDT under a single near‐infrared (NIR) laser (660 nm), resulting in excellent anti‐tumor capacities. Reproduced with permission.^[^ ^73^ ^]^ Copyright 2021, Elsevier. (B) CuS NPs and indocyanine green (ICG) utilized in PTT/PDT accelerating O_2_ production from MnO_2_ in the tumor microenvironment. Reproduced with permission.^[^ ^78^ ^]^ Copyright 2020, Elsevier.

Combining CuS‐catalyzed ROS production and PDT‐effect materials could increase the therapeutic value. Using PDT generating ^1^O_2_, PTT/PDT was jointly achieved by linking BODIPY in the outer layer of CuS@COF.^[^ ^70^ ^]^ FA combined with either CuS NPs or black phosphorus (BP) nanosheets could enhance the therapeutic efficacy of PTT/PDT. When used in conjunction with PTT, NIR laser irradiation could convert O_2_ into ^1^O_2_, thereby suppressing tumors.^[^ ^71^ ^]^ After exposure to 650‐nm laser irradiation to produce ^1^O_2_ in PDT, CuS NPs linked to a porphyrin metal‐organic backbone and infused with ICG could enhance the PTT effect.^[^ ^72^ ^]^ In the presence of a single NIR laser (660 nm), PTT/PDT would be accomplished using CuS NPs and chlorin e6 (Ce6) in CuS@Carbon. Incorporating a carbon shell around CuS NPs could considerably improve their PTT at 660 nm. Under 660‐nm laser irradiation in PDT, Ce6 also effectively suppressed tumor growth (Figure 6A).^[^ ^73^ ^]^

With the addition of the photosensitizer, Ir‐2, CuS NPs were found to develop in situ in MSN nano‐channels, demonstrating extremely high ^1^O_2_ production efficiency during light irradiation, resulting in PTT with excellent results, even with low power irradiation.^[^ ^74^ ^]^ In another research, MSN was used to assemble CuS NPs and g‐C_3_N_4_ quantum dots onto UCNPs. By overlapping their UV and visible light emission peaks with the UV absorption peaks of g‐C_3_N_4_, UCNPs excited by NIR light enabled a synergistic PTT/PDT.^[^ ^75^ ^]^ In addition to achieving the synergistic effect of photosensitizer Ce6 for tumor treatment, lanthanide‐doped UCNPs encapsulated in MSN would offer temperature measurement feedback on CuS‐based PTT.^[^ ^76^ ^]^

The inherently hypoxic microenvironment of tumors and the non‐specific dispersion of photosensitizers reduce the efficacy of PDT. Ce6‐CuS/MSN@PDA@MnO_2_‐FA NPs, which used PDA to encapsulate CuS NPs and Ce6, were activated by PTT/PDT under 808/660 nm laser irradiation to create O_2_ in the tumor microenvironment, resulting in almost total elimination of tumors in tumor‐bearing mice within 2 weeks.^[^ ^77^ ^]^ In a similar study, CuS NPs and ICG were reportedly encased in HA‐coated honeycomb MnO_2_ NPs. Accelerated O_2_ synthesis from MnO_2_ in the tumor microenvironment was achieved using PTT, thereby curing tumor hypoxia. ICG caused a PDT effect and enabled simultaneous imaging with fluorescence and Mn^2+^‐based T_1_‐weighted MR (Figure 6B).^[^ ^78^ ^]^

Many scientists have investigated the tumor death pathways of PTT/PDT. CuS‐MnS_2_ nanoflowers have been studied in preliminary research by Chen et al. for tissue heating and ROS production under NIR laser irradiation, leading to cell necrosis.^[^ ^79^ ^]^ CuS‐NiS_2_ triggered the apoptosis of human gastric cancer cells through the classic Bcl‐2/Bax routes and the novel MLKL/CAPG‐mediated necroptosis mechanisms.^[^ ^80^ ^]^ Further research revealed that PTT/PDT with CuS nanocrystal‐modified Gd‐doped NPs would trigger tumor cell death by attacking the mitochondrial transmembrane potential and causing a change in the levels of anti‐apoptotic (Bcl‐2) and pro‐apoptotic (Bax) proteins, leading to ROS production. The mitochondrial permeability transition pores opened and triggered the caspase‐9/caspase‐3‐dependent apoptotic pathways, enabling more cytochrome c to leak into the cytoplasm.^[^ ^15^ ^]^

#### CDT

**FIGURE 7:**
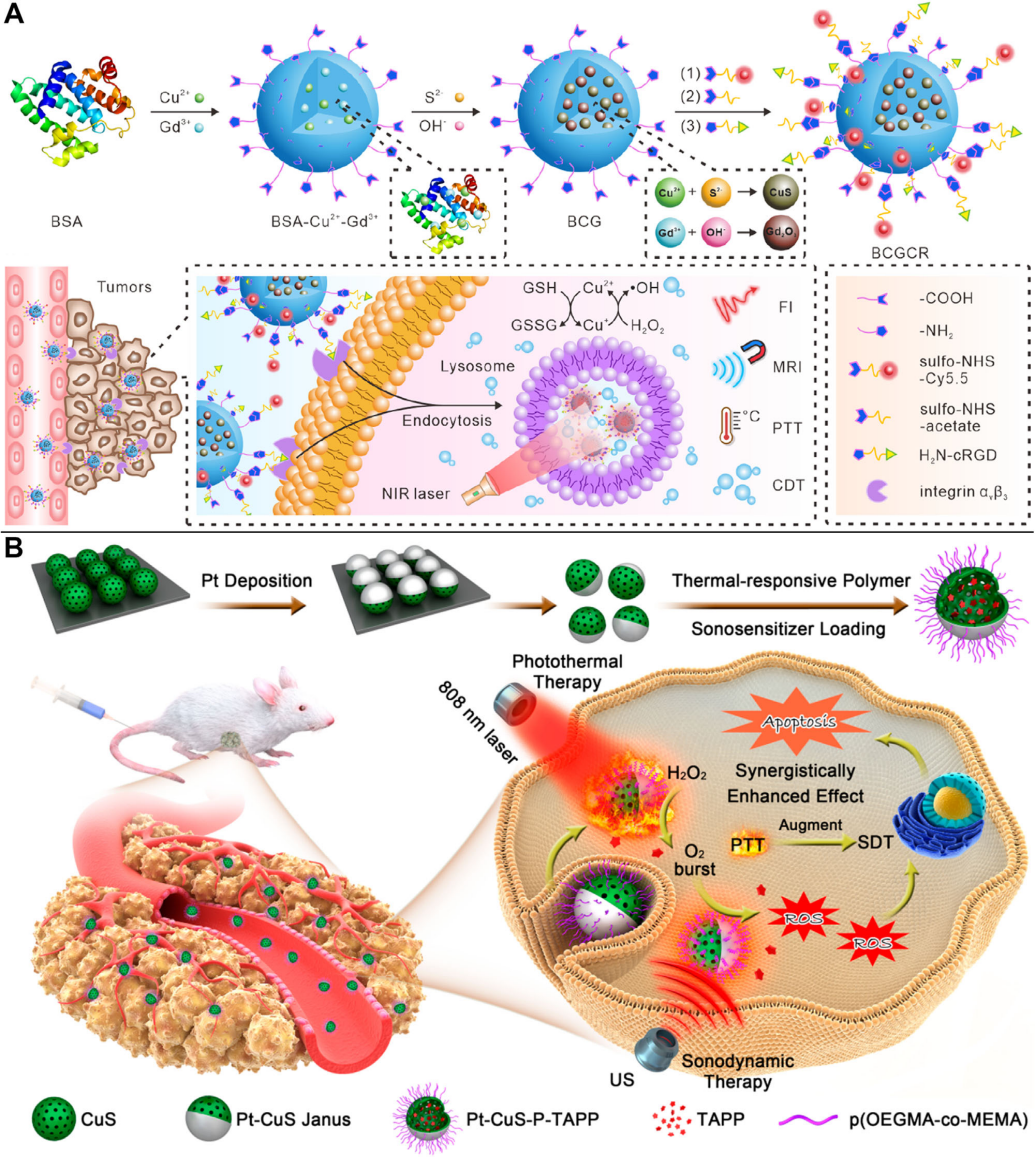
Copper sulfide (CuS)‐based photothermal therapy (PTT) combined with chemodynamic therapy (CDT) or sonodynamic therapy (SDT). (A) More CuS/Gd_2_O_3_ nanoparticles (NPs) internalized in tumor cells resulting in CDT enhancement through a Cu‐induced Fenton‐like reaction. Reproduced with permission.^[^ ^82^ ^]^ Copyright 2022, American Chemical Society. (B) Tetra‐(4‐aminophenyl) porphyrin (TAPP) loaded in Pt‐CuS Janus maximizing the effectiveness of SDT without tumor recurrence, meanwhile PTT regulating TAPP release and enabling Pt to catalyze endogenous H_2_O_2_ to create O_2_ to relieve tumor hypoxia. Reproduced with permission.^[^ ^88^ ^]^ Copyright 2019, American Chemical Society.

CuS NPs are excellent CDT reagents that kill tumor cells because they generate •OH via a Fenton‐like reaction. Enhancing the PCE and CDT effect of CuS NPs has been discovered to be possible via doping with additional metals. In particular, doping Ag into CuS NPs has been demonstrated to significantly increase NIR absorbance and improve their PCE. Silver and copper, which are members of group IB, have similar chemical and physical characteristics. Owing to the small band gap of Ag_2_S nanomaterials, they are useful as NIR fluorescent reagents, whereas the abundance of Cu vacancies in CuS NPs enabled Ag‐Cu doping to improve the synergistic therapeutic effect of CDT.^[^ ^81^ ^]^ Cy5.5 fluorophores and the targeted ligand RGD were used to label CuS/Gd_2_O_3_ NPs stabilized by BSA. The enhanced CDT was further improved by targeting tumor cells to increase the concentration of NPs (Figure 7A). The effectiveness of CDT might be enhanced indirectly using this method.^[^ ^82^ ^]^ Alternatively, CuS NPs were used as catalytic nanoenzymes assembled with the 5th generation poly(amidoamine) dendrimer for the Fenton‐like reaction and linked to glucose oxidase (GOx), which generated H_2_O_2_ and killed cancer cells by increasing the production of •OH. In contrast, PTT could inhibit tumor recurrence and metastasis.^[^ ^83^ ^]^ Additional steps included targeting CD44 receptors with HA‐coated nanocarriers.^[^ ^84^ ^]^ Alternatively, technological improvements for the treatment of tumors involved encapsulating the hydrophilic azo initiator, 2,2′‐azobis(2‐methylpropionamidine) dihydrochloride (AIBA), in CuS NPs and using PTT to initiate thermal decomposition of AIBA, which in turn generated cytotoxic radicals, ultimately driving the release of Cu^2+^ to generate hydroxyl radicals •OH.^[^ ^85^ ^]^ The autocrine and paracrine factors of tumor growth and proliferation in colon cancer include the use of endogenous bioproducts and dysregulated H_2_S generation from the enzymatic system of overexpressed cystathionine β‐synthase. Therefore, to achieve efficient colon cancer treatment, PTT/CDT could be synergistically mediated by endogenous H_2_S‐activated Cu‐MOF nanoenzymes.^[^ ^86^ ^]^

#### SDT

The effectiveness of SDT suffers significantly when O_2_ levels are low. The sonosensitizer tetra‐(4‐aminophenyl) porphyrin (TAPP) encapsulated in hollow CuS NPs with high PCE could overcome tumor hypoxia and improve the effect of SDT, which led to the efficient induction of the apoptosis of tumor cells exposed to ultrasound and the ablation of tumor cells exposed to laser irradiation.^[^ ^87^ ^]^ Further, a new Pt‐CuS Janus NP made of hollow CuS and precious metal Pt was synthesized by Liang et al. Hollow CuS was employed to load the sonosensitizer, TAPP, for SDT into its interior cavity. TAPP release could be regulated by PTT, enabling Pt to catalyze endogenous H_2_O_2_ to create O_2_ and so maximize the efficacy of SDT without tumor recurrence (Figure 7B).^[^ ^88^ ^]^

### 3.4 Immunotherapy

**FIGURE 8:**
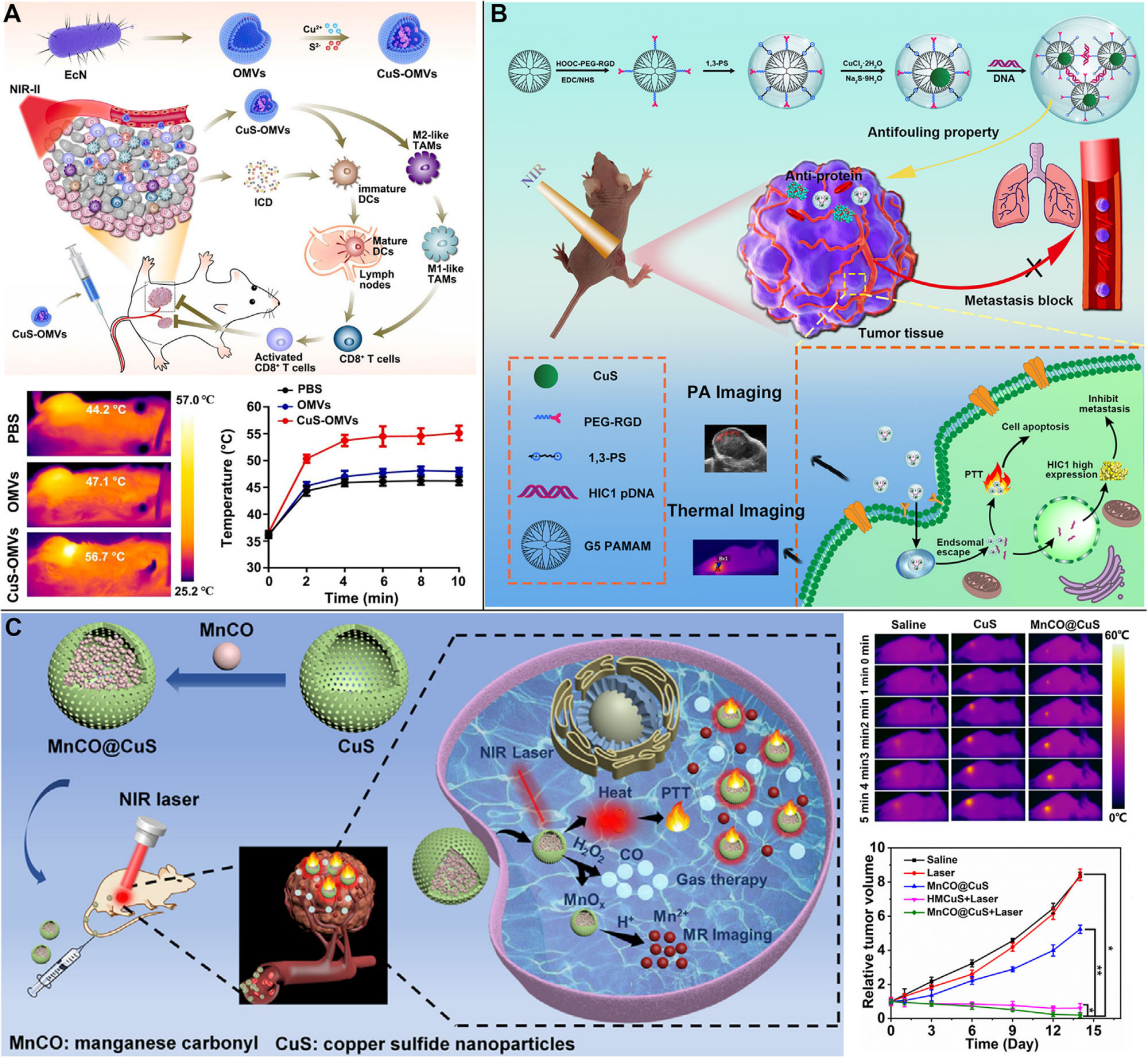
Copper sulfide (CuS)‐based photothermal therapy (PTT) combined with immunotherapy, gene therapy, or gas therapy. (A) Strong immunogenic cell death of tumor promoting dendritic cell maturation and subsequent CD8^+^ T cell activation and repolarizing M2‐like tumor‐associated macrophages to an M1‐like phenotype to remodel the immunosuppressive tumor microenvironment in CuS‐based PTT. Reproduced with permission.^[^ ^90^ ^]^ Copyright 2022, Elsevier. (B) Delivering pDNA of the hypermethylation in cancer 1 gene into cells to prevent tumor cell invasion and lung metastasis in combination with PTT. Reproduced with permission.^[^ ^96^ ^]^ Copyright 2021, American Chemical Society. (C) Hollow mesoporous CuS nanoparticles (NPs) deliver MnCO to release CO and Mn^2+^ in PTT in response to H_2_O_2_ in the tumor microenvironment or near‐infrared (NIR) light stimulation. Reproduced with permission.^[^ ^98^ ^]^ Copyright 2021, Elsevier.

Immunotherapy has been a revolutionary approach in oncology, and the number of PTT/immunotherapy regimens based on CuS NPs has increased in recent years (Table 4 and Table S4). For example, ovalbumin was released more quickly after PTT from CuS NPs, thereby activating CD8^+^ T cells and increasing the levels of IL‐6, IL‐12, and TNF‐α.^[^ ^89^ ^]^ Strong immunogenic cell death of tumors was induced using encapsulating CuS NPs in vesicles made from the outer membranes of *Escherichia coli* Nissle 1917 in PTT. This result led to the activation of dendritic cells, which in turn activated CD8^+^ T cells and the repolarization of M2‐like tumor‐associated macrophages to an M1‐like phenotype, which helped alter the immunosuppressive tumor microenvironment (Figure 8A).^[^ ^90^ ^]^

**TABLE 4:** Copper sulfide (CuS)‐based photothermal therapy (PTT) combined with immunotherapy, gene therapy, and gas therapy.

Names of CuS‐based nanoplatforms	Target ligands	Therapies	Tumor cell lines	Drugs	DEE (%)	DLC (%)	PCE (%)	Lasers used in vitro	Lasers used in vivo	Ref.
CuS@OVA‐PLGA‐NPs	/	Immunotherapy	4T1	Ovalbumin	94.64	4.91	27.9	980 nm, 1.0 W cm^−2^, 5 min	980 nm, 1.0 W cm^−2^, 10 min	[89]
CuS‐OMVs	/	Immunotherapy	4T1	/	/	/	61.4	1064 nm, 0.76 W cm^−2^, 5 min	1064 nm, 1.0 W cm^−2^, 10 min	[90]
FITC‐CuS‐Ab NPs	Cetuximab	Immunotherapy (antibody therapy)	4T1	Cetuximab	/	/	/	1064 nm, 0.2 W cm^−2^, 10 min	1064 nm, 0.2 W cm^−2^, 10 min	[95]
RGD‐CuS DENPs	RGD	Gene therapy	MDA‐MB‐231	/	/	/	49.8	1064 nm, 0.6 W cm^−2^, 5 min	1064 nm, 0.6 W cm^−2^, 5 min	[96]
CuS‐PEI/NO‐TPP	TPP targeting mitochondria	Gas therapy	4T1	NO	/	/	808 nm: 27.37, 1064 nm: 41.80	1064 nm, 1.0 W cm^−2^, 5 min	1064 nm, 1.0 W cm^−2^, 5 min	[97]
MnCO@CuS	/	Gas therapy	MV3	MnCO	48.5	/	40.3	808 nm, 1.0 W cm^−2^, 5 min	808 nm, 1.0 W cm^−2^, 5 min	[98]

Anti‐PD‐1 checkpoint blockade treatment and PTT induced by CuS NPs could eradicate primary tumors and suppress their metastasis.^[^ ^91^ ^]^ Combining immune checkpoint blockade treatment (anti‐PD‐L1) and PTT with maleimide PEG‐modified CuS NPs with tumor antigen adsorption capacity enhanced the serum levels of inflammatory cytokines and the amount of tumor‐infiltrating CD8^+^ T cells.^[^ ^92^ ^]^

PTT in the NIR‐II region can act as a therapeutic method for tumors and significantly induce systemic immune responses, such as the promotion of dendritic cell maturation, CD8^+^ T cell proliferation and infiltration, and elimination of potential metastatic lesions through abscopal effect, by integrating CuS NPs with plasmids encoding IL‐12 genes for gene transfection.^[^ ^93^ ^]^ Tumor immunotherapy was engineered more effective using CuS surface modifications to target Cas9 ribonucleoprotein (RNP) of protein tyrosine phosphatase non‐receptor type 2 (PTPN2), resulting in PTPN2 depletion. This result enabled for the accumulation of infiltrating CD8 T lymphocytes in tumor‐bearing mice and the upregulation of IFN‐ᵧ and TNF‐α expression levels in tumor tissues. This action further improved the anticancer effectiveness when combined with PTT‐induced tumor ablation and immunogenic cell death.^[^ ^94^ ^]^ Collectively, these treatments succeeded in halting the primary tumor growth, as well as mirrored the key benefits of immunotherapy, that is, prevention of metastasis.

Antibody therapy is a type of immunotherapy that involves the use of antibodies that bind specifically to certain cells or proteins. Li et al. modified cetuximab monoclonal antibodies on CuS NPs to increase drug internalization into tumor cells and decrease NIR laser irradiation intensity, resulting in the increased accumulation of CuS‐Ab NPs in the tumor regions and a decrease in cytotoxicity and PTT side effect in normal tissues. Notably, cetuximab has therapeutic potential by targeting EGFR that is abundantly expressed in tumor cells to restrict tumor feeding and ultimately cause cell death.^[^ ^95^ ^]^

### 3.5 Other therapies

#### Gene therapy

Tumor gene therapy is the introduction of certain nucleic acids into cancer cells to ultimately inhibit the production of cancer‐associated proteins. The hypermethylation in cancer 1 gene pDNA was successfully delivered into cells by covalently linking 5th generation poly(amidoamine) dendrimer macromolecules to RGD through PEG to actively target and prevent tumor cell invasion and lung metastasis in combination with PTT (Figure 8B).^[^ ^96^ ^]^

#### Gas therapy

To overcome inherent resistance of tumor cells to heat, intensive PTT could be achieved even in mildly heated conditions and release gas, such as NO, from nanoplatforms to inhibit the expression of heat shock protein HSP90.^[^ ^97^ ^]^ A comparable example comprised a novel hollow mesoporous CuS NP containing MnCO, which could release CO and Mn^2+^ in response to H_2_O_2_ in the tumor microenvironment or NIR light stimulation and be used in T_1_‐weighted MR imaging‐guided PTT (Figure 8C).^[^ ^98^ ^]^

Herein, we opted to introduce the combination of PTT and single therapy. Notably, a different therapeutic modality with PTT will be more efficient in the heating‐related mechanism. Additionally, a novel heating technique is MHT. For instance, a novel three‐material inorganic heterostructure named Fe_3_O_4_@Au@Cu_2‐x_S was created by Fiorito et al. for PTT, MHT, and ^64^Cu radio‐insertion treatment. The heterostructure had a higher heating efficiency; however, further investigation on in vivo synergetic therapy should be explored.^[^ ^99^ ^]^ Nonetheless, MHT overlaps with the effect caused by PTT; therefore, the number of such studies is limited.

## 4 CuS‐BASED PTT COMBINED WITH TWO OR MORE THERAPIES

Table 5 and Table S5 summarize the nanoplatforms employed in CuS‐based PTT in combination with two or more treatments.

**TABLE 5:** Copper sulfide (CuS)‐based photothermal therapy (PTT) combined with two or more therapies.

Names of CuS‐based nanoplatforms	Target ligands	Therapies	Tumor cell lines	Drugs	DEE (%)	DLC (%)	PCE (%)	Lasers used in vitro	Lasers used in vivo	Ref.
PTX@CuS@MMNPs	Cell membrane, iRGD	Chemo/PDT	4T1	PTX	∼90	∼100	54	808 nm, 1.0 W cm^−2^, 5 min	808 nm, 1.0 W cm^−2^, 5 min	[100]
H‐CuS@PCM/DOX/Ce6 (HPDC) NPs	/	Chemo/PDT	4T1	DOX, Ce6	DOX: 84.9, Ce6: 85.2	DOX: 6.98, Ce6: 17.94	44.13	660 nm, 0.5 W cm^−2^, 5 min; 808 nm, 2.0 W cm^−2^, 5 min	660 nm, 0.5 W cm^−2^, 5 min; 808 nm, 2.0 W cm^−2^, 5 min	[102]
B/USCs‐PEG‐DOX	/	Chemo/PDT	HeLa, U14	DOX	77.4	/	30.3	808 nm, 1.0 W cm^−2^, 10 min	808 nm, 1.0 W cm^−2^, 10 min	[103]
DOX/CuS@Cu‐MOF/PEG	/	Chemo/CDT	4T1	DOX	57.2	25.5	39.6	1064 nm, 1.0 W cm^−2^, 2–8 min	1064 nm, 1.0 W cm^−2^, 10 min	[104]
CuS@mSiO_2_@MnO_2_/DOX	/	Chemo/CDT	HeLa	DOX	/	/	40.49	915 nm, 1.0 W cm^−2^, 5 min	915 nm, 1.0 W cm^−2^, 10 min	[105]
CuS‐RNP/DOX@PEI	/	Chemo/gene therapy	A375, MCF‐7, HeLa	DOX	18.85	/	/	808 nm, 2.0 W cm^−2^, 6 and 10 min	808 nm, 2.0 W cm^−2^, 6 and 10 min	[106]
COPIRS&Dox@PhPP NPs	/	Chemo/gas therapy	4T1	DOX, CO	/	CO: 0.92; DOX: 4.82	/	980 nm, 0.78 W cm^−2^, 10 min	980 nm, 0.78 W cm^−2^, 20 min	[107]
GOx‐Gd‐CuS@MSNs	/	PDT/CDT	4T1	GOx	/	Gd: 1.2; GOx: 41.80	30.8	808 nm, 1.5 W cm^−2^, 5 min	808 nm, 1.5 W cm^−2^, 5 min	[109]
AIPH/PDA@CuS/ZIF‐8	/	PDT/CDT	4T1	AIPH	49.8	20.75	28.05	1064 nm, 1.0 W cm^−2^, 10 min	1064 nm, 1.0 W cm^−2^, 10 min	[110]
IONF@CuS	/	PDT/MHT	PC3	/	/	/	42	1064 nm, 0.1 or 0.3 W cm^−2^, 5 min	1064 nm, 1.0 W cm^−2^, 10 min	[112]
FA‐CD@PP‐CpG	FA	Chemo/PDT/immune‐therapy	4T1	DTX	/	23	/	650 nm, 4.5 mW cm^−2^; 808 nm, 0.987 W cm^−2^, 5 min	650 nm, 4.5 mW cm^−2^; 808 nm, 0.987 W cm^−2^, 5 min	[113]

### 4.1 Chemotherapy and PDT

Typically, CuS NPs are utilized as heat sources for releasing loaded chemotherapeutic drugs, with the photothermal effect serving as the mechanism of action. To exploit the PTT action of CuS NPs, chemotherapeutic drugs are often injected into the cavity of hollow mesoporous CuS NPs or co‐loaded with them. In contrast, PDT is associated with two primary approaches: (1) using the PDT effect of CuS NPs directly; (2) loading the photosensitizers possessing the PDT effect.

**FIGURE 9:**
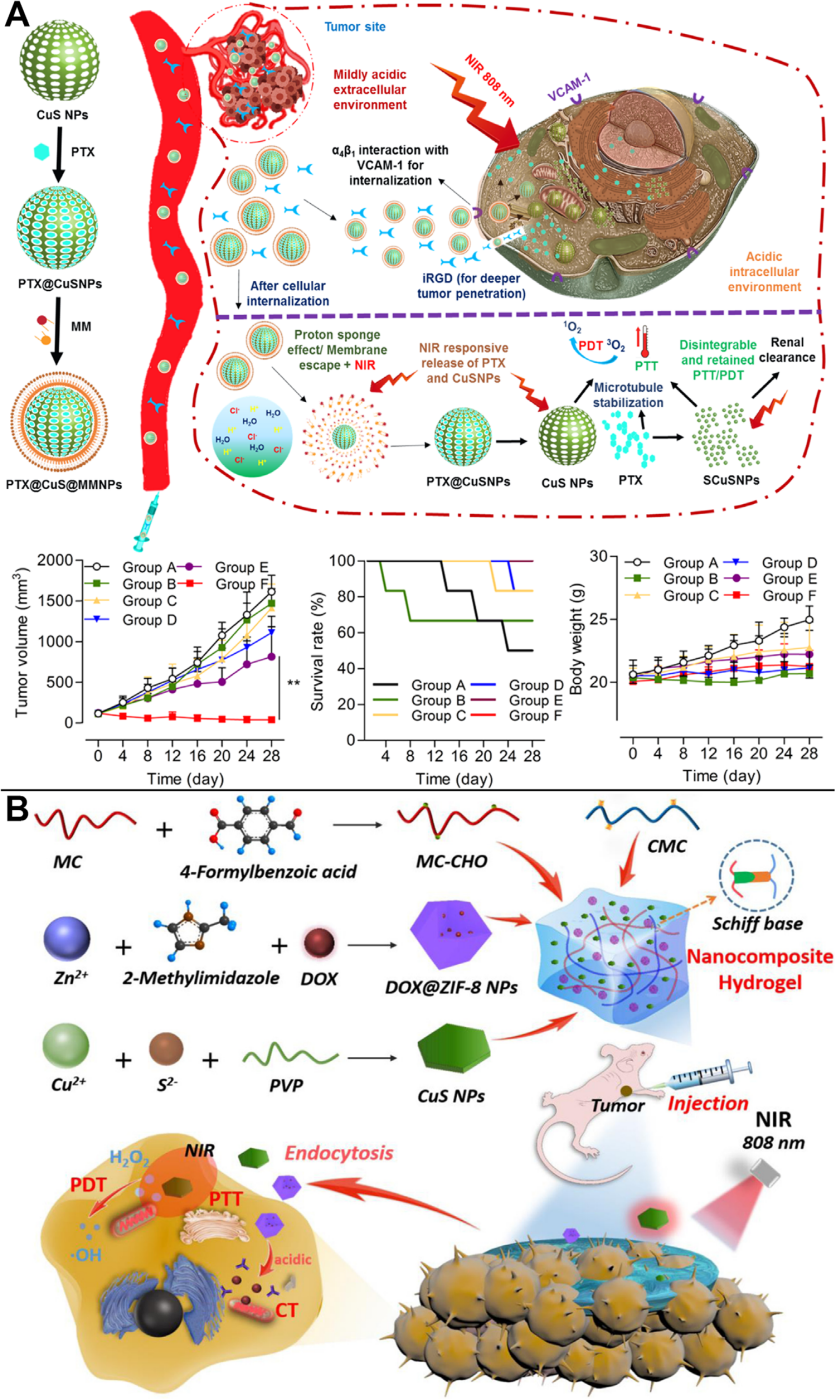
Copper sulfide (CuS)‐based photothermal therapy (PTT) combined with chemo/photodynamic therapy (PDT). (A) Synergistic chemo/PTT/PDT achieved using PTX@CuS@MMNPs owing to their enhanced active targeting and cell internalization capabilities, as well as performing PTT, releasing paclitaxel (PTX), and converting O_2_ to ^1^O_2_. Reproduced with permission.^[^ ^100^ ^]^ Copyright 2020, American Chemical Society. (B) Hydrogels containing CuS and DOX@ZIF‐8 NPs converting O_2_ into toxic reactive oxygen species (ROS), and the acidic tumor microenvironment facilitating the decomposition of DOX@ZIF‐8 NPs, leading to the regulated release of doxorubicin (DOX). Reproduced with permission.^[^ ^101^ ^]^ Copyright 2022, Elsevier.

PTT derived using CuS was primarily administered to release chemotherapeutic drugs or photosensitizers in these situations. However, the PDT used in ternary combination treatment was generated by distinct processes, such as CuS NPs inherently serving as PDT agents. Active targeting capabilities and cell internalization may also be enhanced, for instance, by PTX@CuS@MMNPs equipped with macrophage membranes and the ligand, 9‐amino acid cyclic peptide iRGD, for camouflage. Synergistic chemo/PTT/PDT could be generated using laser irradiation by performing PTT, releasing PTX, and converting O_2_ to ^1^O_2_ simultaneously (Figure 9A).^[^ ^100^ ^]^ Qian et al. used hydrogels to distribute CuS NPs and DOX@ZIF‐8 NPs. ROS were created from O_2_ using the PDT produced by CuS NPs upon exposure to NIR light. The acidic tumor microenvironment might facilitate the decomposition of DOX@ZIF‐8 NPs, leading to the regulated release of DOX (Figure 9B).^[^ ^101^ ^]^

Other PDT agents, such as the widely used photosensitizer, Ce6, have often been utilized. PTT could cause the regulated release of DOX and Ce6 from a phase change material (PCM, 1‐tetradecanol) encapsulated inside hollow CuS NPs. Tumors were subsequently eradicated owing to the complementary action of DOX chemotherapy and Ce6 PDT.^[^ ^102^ ^]^ To accomplish synergistic chemo/PTT/PDT, BP nanosheets could anchor DOX on UCNPs, and both BP nanosheets and UCNPs could be coated in MSN. If exposed to light, BP nanosheets could produce ROS. With 808‐nm laser irradiation, UCNPs could offer multimodal CT/MR imaging, overcoming the drawbacks of poor efficiency and restricted penetration depth of UV/visible light.^[^ ^103^ ^]^ Overall, combining chemotherapy with PDT is a typical strategy as it increases the effectiveness of both treatments against tumors.

### 4.2 Chemotherapy and CDT

**FIGURE 10:**
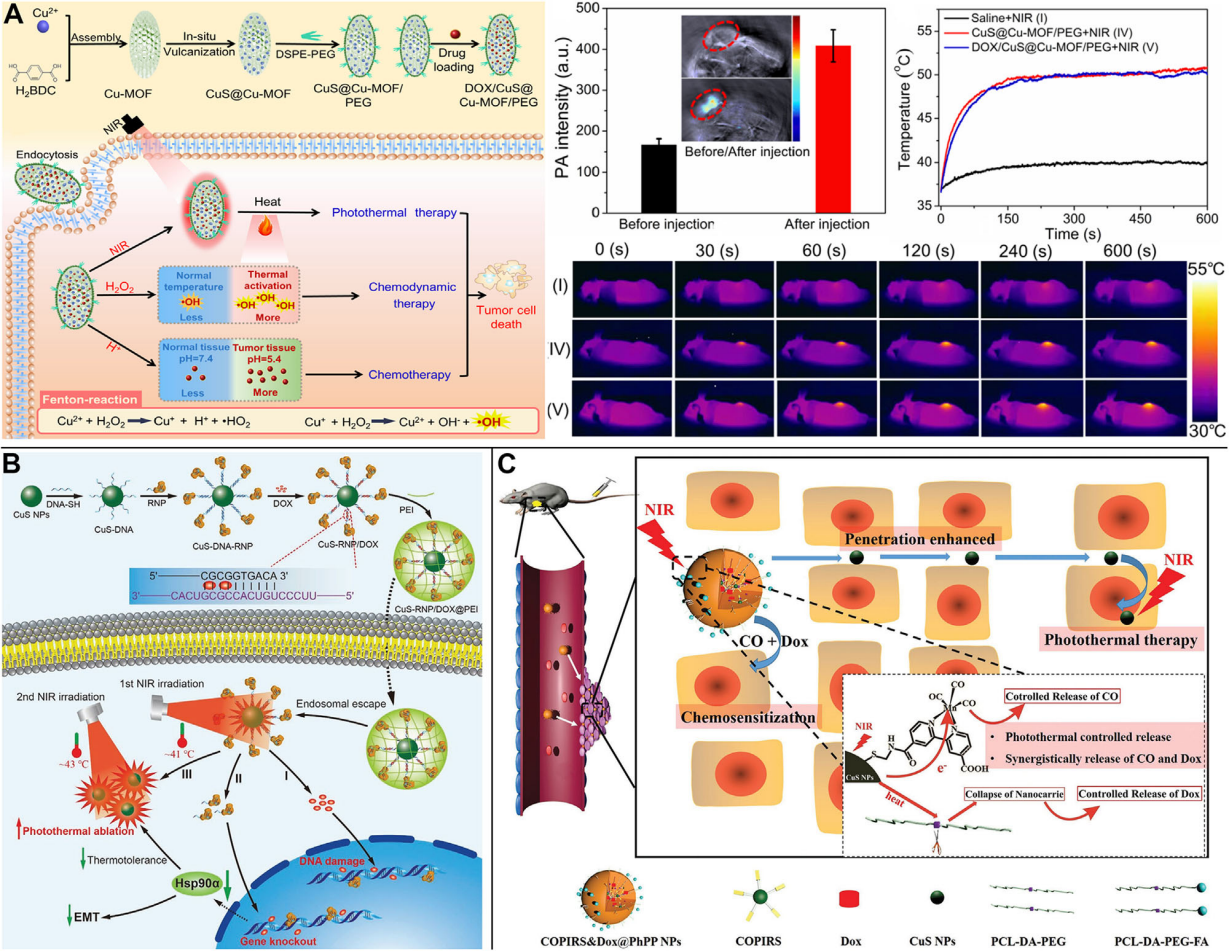
Copper sulfide (CuS)‐based photothermal therapy (PTT) combined with chemo/chemodynamic therapy (CDT), chemo/gene therapy, or chemo/gas therapy. (A) During CDT, CuS/DOX@Cu‐MOF/PEG creating •OH in a Fenton‐like reaction, realizing doxorubicin (DOX) release for chemotherapy, PA imaging, and PTT. Reproduced with permission.^[^ ^104^ ^]^ Copyright 2022, Elsevier. (B) CRISPR‐Cas9 RNP and DOX controlled release for combined chemo/gene therapy with decreasing tumor heat resistance and increasing the efficacy of the hypothermic PTT. Reproduced with permission.^[^ ^106^ ^]^ Copyright 2021, John Wiley & Sons. (C) A synergistic chemo/gas therapeutic nanoplatform co‐releasing CO and DOX from CuS nanoparticles (NPs) during PTT.^[^ ^107^ ^]^ Copyright 2022, John Wiley & Sons.

A synergistic effect was demonstrated among the primary chemotherapeutic agent DOX, CDT (which employed Fenton‐like processes to create •OH), and PTT in tumor cells. For example, the COF multifunctional nanoplatform CuS@COFs‐BSA‐FA/DOX was employed for synergistic chemo/PTT/CDT, in addition to serving as a fluorescence probe and DOX delivery nanocarrier. Tumor apoptosis could be triggered by combining an acidic tumor microenvironment, NIR light, and PTT‐induced local thermotherapy.^[^ ^67^ ^]^ As a chemotherapeutic drug, DOX could be encapsulated in CuS@Cu‐MOF, whereas PTT with Cu‐MOF employed 4‐nm CuS NPs and generated •OH in CDT through a Fenton‐like reaction (Figure 10A).^[^ ^104^ ^]^ Using CuS‐mediated PTT and O_2_ production from GSH‐triggered H_2_O_2_ and toxic •OH catalyzed by MnO_2_, CuS@mSiO_2_@MnO_2_ nanocomposites alleviated tumor hypoxia and improved CDT in the acidic tumor microenvironment. Simultaneously, T_1_‐weighted MR imaging with the produced Mn^2+^ could be achieved.^[^ ^105^ ^]^ These combined chemo/PTT/CDT treatments can markedly slow tumor development.

### 4.3 Chemotherapy and gene therapy

The regulated release of CRISPR‐Cas9 RNP and DOX using CuS NPs facilitated a synergistic chemo/gene therapy. CuS NP‐linked DNA fragments and single‐strand RNA were employed as regulatory elements for the photothermal activation of a gene‐editing unit and DOX release. Cas9 RNP lowered the levels of Hsp90α, a heat shock protein, which decreased tumor heat resistance and increased the efficacy of the hypothermic PTT (Figure 10B).^[^ ^106^ ^]^

### 4.4 Chemotherapy and gas therapy

The development of a synergistic chemo/gas therapeutic nanoplatform relied on the ability to co‐release CO and DOX from CuS NPs. The combination of PTT and chemotherapeutic sensitization could eliminate active tumor cells. Further, the disassembly of the nanoplatform led to the release of CuS NPs. Owing to their small size, CuS NPs could penetrate solid tumors more deeply, and NIR laser irradiation further enhanced PTT, eliminating the tumors quickly and efficiently. To successfully kill tumor cells, the cascade multi‐stage treatment technique of “PTT + chemotherapy sensitization” was used (Figure 10C).^[^ ^107^ ^]^

### 4.5 PDT and CDT

**FIGURE 11:**
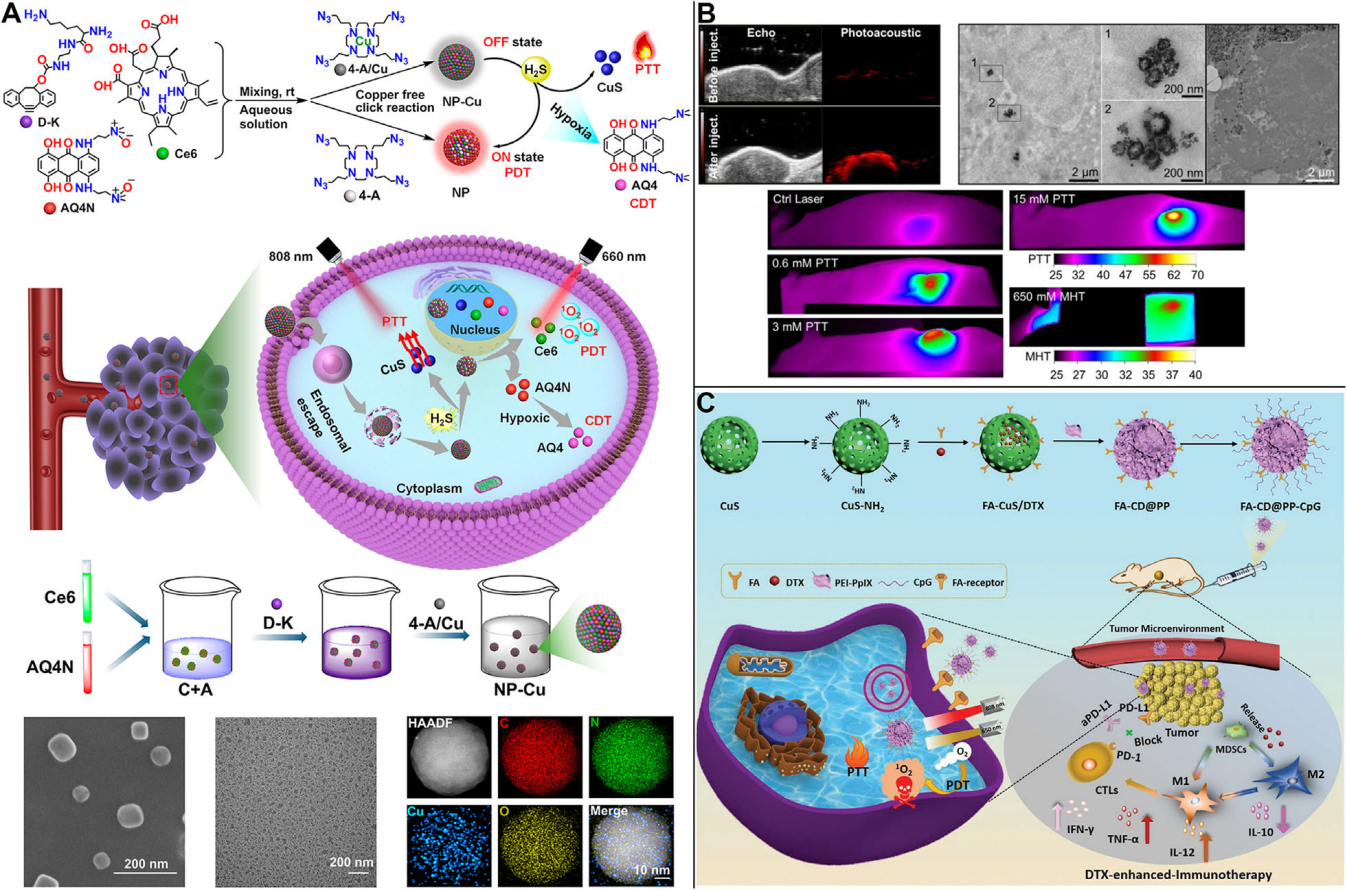
Copper sulfide (CuS)‐based photothermal therapy (PTT) combined with PDT/CDT, PDT/MHT, or chemo/PTT/PDT/immunotherapy. (A) Synergistic PTT/PDT/CDT intensified by the light cascade, as tumor‐derived H_2_S synthesizing CuS for PTT, Ce6 releasing for PDT, and exacerbating intracellular hypoxic stress to induce AQ4N‐associated CDT. Reproduced with permission.^[^ ^111^ ^]^ Copyright 2022, American Chemical Society. (B) CuS nanoflower‐like nanoparticles (NPs) with γ‐Fe_2_O_3_ core and spikes on the surface used in PTT/PDT/MHT with magnetic resonance (MR) and photoacoustic (PA) imaging.^[^ ^112^ ^]^ Copyright 2019, Ivyspring International Publisher. (C) Synergistic chemo/PTT/PDT/immunotherapy with a multifunctional nanocomposite to improve the anti‐tumor efficacy. Reproduced with permission.^[^ ^113^ ^]^ Copyright 2019, John Wiley & Sons. PDT, photodynamic therapy; CDT, chemodynamic therapy; MHT, magnetic hyperthermia.

Similar to earlier combination models, GOx‐encapsulated CuS NPs could consume glucose, resulting in tumor starvation, whereas the generated H_2_O_2_ contributed to synergistic PTT/PDT/CDT. Further, the Fenton‐like reaction generated endogenous chloride, resulting in tumor death^[^ ^108^ ^]^ or tumor cell apoptosis.^[^ ^109^ ^]^ Similarly, 2,2′‐azobis[2‐(2‐imidazolin‐2‐yl)propane] dihydrochloride (AIPH) was loaded in PDA@CuS/ZIF‐8 for PA imaging. GSH and H_2_O_2_ depletion under laser irradiation generated •OH in CDT, and AIPH decomposition generated hazardous alkyl radicals (•R) in PDT, resulting in total tumor elimination in 14 days.^[^ ^110^ ^]^ A light cascade‐enhanced synergistic PTT/PDT/CDT was achieved by Yang et al. using the cyclen‐Cu^2+^ complexes, where tumor‐derived H_2_S reacted with Cu^2+^ to generate CuS for PTT and release Ce6 for PDT. The intracellular hypoxic stress was amplified to trigger AQ4N‐associated CDT, resulting in a light cascade‐enhanced synergistic PTT/PDT/CDT. Notably, the use of hypoxia‐activated prodrugs has been considered as a chemotherapy option (Figure 11A).^[^ ^111^ ^]^

### 4.6 PDT and MHT

Curcio et al. developed CuS nanoflower‐like NPs with a γ‐Fe_2_O_3_ core and spikes on the surface. The use of CuS in PTT and PDT and γ‐Fe_2_O_3_ core in MHT resulted in increased anti‐tumor capacity and decreased dose of NPs, laser exposure power density, and magnetic field frequency in vitro and in vivo, enabling continuous PTT and MHT with monitoring of the treatment process with MR and PA imaging (Figure 11B).^[^ ^112^ ^]^

### 4.7 Chemotherapy, PDT, and immunotherapy

Immunotherapy is not as successful as expected owing to the factors such as low PD‐L1 expression, poor infiltration of cytotoxic T lymphocytes (CTL), and large numbers of myeloid‐derived suppressor cells (MDSCs). To improve immunotherapy, Chen et al. created a multifunctional nanocomposite for synergistic docetaxel (DTX) chemo/PTT/PDT. Under 650/808 nm irradiation, the nanocomposite displayed optimal PDT/PTT efficiency, which significantly suppressed tumor progression in vivo. By promoting CTL infiltration and inhibiting MDSCs while efficiently polarizing MDSCs toward the M1 phenotype to lower tumor burden, low‐dose DTX increased the effectiveness of anti‐PD‐L1 antibody, and ultimately improved the anti‐tumor efficacy (Figure 11C).^[^ ^113^ ^]^

The combination of three therapies, with PTT as one of the therapies, is more successful than the combination of two therapies. Notably, the combination of MHT/PTT/PDT is one of the few instances utilizing CuS NPs of in vivo tumor MHT. More significantly, it is vital to propose novel techniques and test their effectiveness in vivo in the future. Further, better outcomes will be achieved if a unique therapeutic approach is designed for specific biological mechanisms.

## 5 BIOCOMPATIBILITY OF CuS‐BASED PTAS

Researchers have created numerous CuS‐based PTA nanoplatforms used as synergistic therapies, including PTT. Nonetheless, assessing both their in vitro and in vivo biosafety is critical. Currently, in vitro biosafety is mainly associated with cytotoxicity and hemolysis, whereas in vivo biosafety is primarily based on H&E staining of major organs, blood routine analysis, liver and renal functional evaluations, and body weight monitoring. The majority of experiments did not exhibit evident cytotoxicity even at concentrations of CuS‐based PTAs as high as 2 mg mL^−1^,^[^ ^25^ ^]^ demonstrating their optimal biosafety in vitro despite variations in the maximal concentrations of CuS‐based PTAs. After a period of injection in vivo, H&E staining revealed no visible inflammation or damage in major organs. Further, the absence of any apparent harm to the liver and renal functions suggested their high‐level biosafety in vivo. However, only some studies were conducted to enable biosafety evaluations, and more biosafety assessments are still need.

Guo et al. evaluated the biotoxicity and metabolism of hollow CuS NPs (HCuSNPs) and hollow gold NPs (HAuNS), which have identical particle sizes, morphologies, and surface modifications of PEG. Approximately 90% of PEG‐HCuSNPs could be eliminated one month after intravenous injection (approximately 67% through hepatobiliary excretion and 23% through renal excretion); however, only 3.98% of total PEG‐HAuNS excretion was demonstrated, indicating that PEG‐HCuSNPs were considered biodegradable and their metabolization primarily depended on the hepatobiliary system. Additionally, few PEG‐HCuSNPs were converted into small CuS NPs within 12 h, which could be eliminated by the kidneys. A significant period (12 h–1 month) might pass before residual PEG‐HCuSNPs are metabolized into Cu^2+^ and eliminated via the urine. Neither histological nor blood biochemical analysis revealed any apparent toxicity after injection. Further, a reversible change of proteins was observed after PEG‐HCuSNPs injection. In contrast, an irreversible change of proteins was observed in the PEG‐HAuNS group, with elevated serum lactate dehydrogenase at 3 months, indicating potential long‐term toxicity of HAuNS. These findings demonstrate that HCuSNPs are biodegradable and biocompatible.^[^ ^114^ ^]^

Feng et al. thoroughly investigated the toxicity of CuS nanoplates both in vitro and in vivo. The increased CuS nanoplate concentrations were cytotoxic to HUVEC and RAW264.7 cells, although low concentrations had no noticeable side‐effect on the cytoskeleton. The acute toxicity revealed that the maximum tolerated dose and LD_50_ were 8.66 and 54.5 mg kg^−1^, respectively. CuS nanoplates were primarily excreted by the hepatobiliary system, the intestine, or urine after accumulation in the liver, spleen, and lungs. The blood biochemistry and tissue section analysis did not reveal pathological inflammation or impaired liver and renal function.^[^ ^115^ ^]^ Zhang et al. evaluated the effect of polymer‐modified CuS nanoclusters (PATA3‐C4@CuS) on zebrafish embryos. When the concentration of PATA3‐C4@CuS was higher than 1 mg L^−1^, aberrant phenotypes and increased mortality became visible. Proteins that are not expressed typically might cause cell migration destruction during gastrulation. As zebrafish larvae had a slower heart rate, smaller ventricles, and impaired ventral projection of the primary motor neurons, PATA3‐C4@CuS exhibited slight toxicity.^[^ ^116^ ^]^

The findings revealed that CuS‐based PTAs did not exhibit apparent cytotoxicity and toxicity in vivo. Further, CuS NPs could be eliminated via the liver and kidneys. According to blood biochemical analysis, CuS‐based PTAs had no side‐effect on the liver and renal functions. Further, no pathological inflammation or other damage was observed in the organ tissue sections stained with H&E. However, as CuS‐based PTAs were toxic to zebrafish embryos, they may affect development and neurons. Therefore, more research is needed to determine the biocompatibility of CuS‐based PTAs.

## 6 CONCLUSIONS AND PERSPECTIVE

In this review, we first highlighted cases of CuS‐based PTT used alone before discussing synergistic treatment methods including chemotherapy, radiotherapy, dynamic therapies (PDT, CDT, and SDT), immunotherapy, gene therapy, gas therapy, and MHT. Therefore, we opted to pay special attention to synergistic treatment with CuS‐based PTT and several therapies. Notably, the applications of CuS‐based PTT can be widened because CuS NPs can be used for molecular imaging,^[^ ^14^ ^]^ and the treatment of diseases other than tumors,^[^ ^117^ ^]^ such as rheumatoid arthritis,^[^ ^118^ ^]^ bacterial infections,^[^ ^119^, ^120^ ^]^ wound healing,^[^ ^121^, ^122^ ^]^ atherosclerosis,^[^ ^123^ ^]^ and arterial restenosis.^[^ ^124^ ^]^ Several studies opted to integrate PTT and imaging methods, which is an effective and promising approach. The paramagnetic Cu(II) in CuS NPs may be utilized in T_1_‐weighted MR imaging. However, the effectiveness is significantly less favorable than that of iron, cobalt, and nickel‐based nanomaterials. PET imaging is possible with the addition of ^64^Cu(II) instead of Cu(II) in CuS, but more significantly, CuS NPs are particularly well‐suited as contrast agents for PA imaging owing to their strong NIR‐I and II absorbances. Multimodal imaging may also be conducted using these imaging approaches. Imaging may be used to assess drug delivery, the progress of therapy, and the effectiveness of CuS NPs‐based PTAs. The light intensity and imaging duration are substantially lower than those for PTT. In addition, the wavelength may be the same or different, which does not affect the results of imaging or PTT. Accordingly, imaging and PTT cannot be performed simultaneously, warranting the development of novel technologies that can combine them.

CuS NPs can be manufactured in the lab using various techniques; however, it is still challenging to precisely regulate the atomic ratio of Cu and S, as well as the size distribution, morphology, and crystal structure of CuS NPs. Strong LSPR in the NIR region is often attributed to a shortage of Cu atoms in CuS crystals, which is directly related to the photothermal characteristics of CuS NPs.^[^ ^12^ ^]^ Consequently, precisely adjusting the atomic ratio of Cu and S to maximize the PCE of CuS NPs is challenging. Scaling up the synthesis of a significant quantity of CuS NPs using synthetic techniques in the lab is similarly problematic from a commercialization standpoint. Furthermore, Cu^2+^ released from CuS NPs has a significant health effect.^[^ ^117^ ^]^ Currently, the biological theories involving Cu^2+^ are not generally understood. Only the Fenton‐like reaction in CDT has been well understood, and further research is needed to determine the long‐term effect on cells, tissues, and organs.

Many unresolved issues remain with CuS‐based PTT, despite the performance of extensive research and more improvements to increase PTT effectiveness. First, most studies focused on the materials, and the animal models were generally straightforward. Second, several assessments were not cited in this review as subsequent studies were not performed after the cell culture studies. Third, the use of 4T1 cell lines to establish in vivo tumor models is widely reported in the literature. Whether the tumor microenvironment in the mouse‐derived 4T1 breast cancer cell line‐based subcutaneous tumor model is comparable with that of human‐derived tumors is a topic of interest. When PTT was combined with chemotherapy, the standard agent was the model chemotherapeutic drug, DOX, which markedly reduced the efficacy of the combined treatment. Although CuS NPs might be physiologically inert and biocompatible, additional studies on their late degradation, integrated nanoplatforms, and biotoxicity investigations are still required.^[^ ^125^ ^]^

When lasers in the NIR‐II region were used, such as 1064 nm, the laser power densities were not uniform, the relationship between power density and time was not explored, and the setting of conditions was more reliant on experience. However, the most common 808‐nm laser was employed in many investigations for light irradiation, with exposure doses exceeding the FDA‐recommended standard (0.33 W cm^−2^).^[^ ^22^, ^23^ ^]^ Nonetheless, a standardized technique is needed to calculate the synergistic efficacy of PTT with other treatments via tumor suppression rate, thereby complicating meaningful comparisons between them. PTT conditions also prevent direct comparisons between values obtained using the same index.

PTT strategies serve as another integral approach that must be studied. For instance, two‐fraction NIR light has been suggested for killing tumor cells.^[^ ^18^, ^107^ ^]^ PTT has been included in phased techniques to avoid tumor metastasis and recurrence after surgery and chemotherapy.^[^ ^83^ ^]^ As this feature is seldom reported and most studies focus on direct synergistic treatment between PTT and other therapies, this feature should be further explored. However, the benefits of PTT in preventing tumor metastasis and recurrence are only partially presented in the literature.

Most publications on this topic were related to materials science. Consequently, the in vitro and in vivo biological effects of the compounds were largely ignored. For example, Deng et al. discovered that CuS@mSiO_2_‐PEG NPs might inhibit tumor cell migration, which might be associated with MMP‐2 and MMP‐9 and closely related to different non‐receptor tyrosine kinase protein/adhesive spot kinase signaling pathways in vivo and in vitro. Notably, CuS@mSiO_2_‐PEG NPs significantly decreased tumor metastasis and increased survival in tumor‐bearing mice.^[^ ^126^ ^]^ These situations are understudied and underrepresented in the literature, ultimately causing the practical application of PTT more challenging and fueling its isolation from clinical medicine and medical devices. Future studies integrating biology and clinical medicine closely are thus warranted.

In conclusion, CuS‐based PTT has excellent potential for curing diseases, which has attracted extensive attention from biological researchers and clinical workers. The synergism between CuS‐based PTT and other therapies can play better role in cancer therapies. Finally, CuS‐based PTT can be included in tumor protocols as a future clinical treatment.

## CONFLICT OF INTEREST STATEMENT

The authors declare no conflict of interest.

## Supporting information

Supporting Information is available from the Wiley Online Library or from the author.
