# Supplementary material for: In vivo synergistic tumor therapies based on copper sulfide photothermal therapeutic nanoplatforms
Source: Exploration (Beijing). 2023 Jun 24;3(5):20220161. doi: 10.1002/EXP.20220161 (PMC10582616; doi:10.1002/EXP.20220161)
Supplement: Supplementary file 1 — Supporting Information is available from the Wiley Online Library or from the author. [file EXP2-3-20220161-s001.docx]

Supporting Information

**In vivo synergistic tumor therapies based on copper sulfide photothermal therapeutic nanoplatforms**

Jingwen Ma^1^, Na Li^1^, Jingjian Wang^1^, Zhe Liu^2^, Yulong Han^3^, and Yun Zeng^4,5^

^1^ Radiology Department, CT and MRI Room, Ninth Hospital of Xi’an, Ninth Affiliated Hospital of Medical College of Xi’an Jiaotong University, Xi’an, Shaanxi Province, P. R. China

^2^ Department of Pathology, Ninth Hospital of Xi’an, Ninth Affiliated Hospital of Medical College of Xi’an Jiaotong University, Xi’an, Shaanxi Province, P. R. China

^3^ School of Engineering and Applied Sciences, Harvard University, Cambridge, Massachusetts, USA

^4^ School of Life Science and Technology, Xidian University and Engineering Research Center of Molecular and Neuro Imaging, Ministry of Education, Xi’an, Shaanxi Province, P. R. China

^5^ International Joint Research Center for Advanced Medical Imaging and Intelligent Diagnosis and Treatment and Xi’an Key Laboratory of Intelligent Sensing and Regulation of trans-Scale Life Information, School of Life Science and Technology, Xidian University, Xi’an, Shaanxi Province, P. R. China

**Correspondence**

Jingwen Ma, Radiology Department, CT and MRI Room, Ninth Hospital of Xi’an, Ninth Affiliated Hospital of Medical College of Xi’an Jiaotong University, Xi’an, Shaanxi Province 710054, P. R. China.

Email: majingwen891031@xjtu.edu.cn

Yun Zeng, School of Life Science and Technology, Xidian University and Engineering Research Center of Molecular and Neuro Imaging, Ministry of Education, Xi’an, Shaanxi Province 710126, P. R. China.

Email: yzeng@xidian.edu.cn

**Abbreviations**

CuS: copper sulfide; PTT: photothermal therapy; PCE: photothermal conversion efficiency; DEE: drug encapsulation efficiency; DLC: drug loading content; PDT: photodynamic therapy; CDT: chemodynamic therapy.

**Table S1.** CuS-based PTT used in vitro and in vivo.

| Names of CuS-based nanoplatforms | Target ligands | Tumor cell lines | PCE (%) | Lasers used in vitro | Lasers used in vivo | Ref. |
| --- | --- | --- | --- | --- | --- | --- |
| CuS-melanin-FA NPs | FA | 4T1, HeLa, U87mg | 29.85 | 808 nm, 1.0 W cm^-2^, 5 min | 808 nm, 1.0 W cm^-2^, 5 min | [16] |
| CuPDF NPs | / | 4T1 | 37.4 | 808 nm, 1.0 W cm^-2^, 10 min | 808 nm, 1.0 W cm^-2^, 10 min | [17] |
| GRS-DNA-CuS | / | A549 | 58.87 | 808 nm, 2.8 W cm^-2^, 5 min | 808 nm, 2.8 W cm^-2^, 2 min | [18] |
| ^68^Ga-Mn-CuS@BSA NDs | / | SKOV-3 | 47.03 | 980 nm, 1.0 W cm^-2^, 3 min | 980 nm, 1.0 W cm^-2^, 3 min | [19] |
| RGD-CuS-Cy5.5 NPs | RGD | MKN45 | 51 | 808 nm, 1.0 W cm^-2^, 10 min | 808 nm, 1.0 W cm^-2^, 10 min | [20] |
| T-MAN | cRGD | MKN45 | 70.1 | 808 nm, 0.8 W cm^-2^, 5 min | 808 nm, 0.85 W cm^-2^,10 min; metastatic LNs: 808 nm, 0.5 W cm^-2^, 10 min | [21] |
| CuS NPs | / | MCF-7 | 42.2 | 1064 nm, 1.0 W cm^-2^, 10 min | 1064 nm, 1.0 W cm^-2^, 10 min | [24] |
| WK@CuS NFs | / | 4T1 | 32.9 | 1064 nm, 1.0 W cm^-2^, 5 min | 1064 nm, 1.0 W cm^-2^, 10 min | [25] |
| PB@PAA/CuS JNPs | / | KB, HepG-2, MCF-7 | 39 | 808+1064 nm, 1.0 W cm^-2^, 5 min | 808+1064 nm, 1.0 W cm^-2^, 5 min | [26] |
| CuS-TPP-HA | HA; TPP targeting mitochondria | MCF-7 | / | 1064 nm, 0.75 W cm^-2^, 5 or 10 min | 1064 nm, 1.0 W cm^-2^, 10 min | [27] |
| CuS-PEG-NPs | / | 4T1 | / | 808 nm, 0.33 W cm^-2^; 1275 nm, 1.0 W cm^-2^, 5 min | 808 nm, 1.0 W cm^-2^; 1275 nm, 1.0 W cm^-2^; 0.2 W cm^-2^, 5 min | [28] |
| CuS@PDA-TDHPs | / | MCF-7, HeLa | 35.12 | 1064 nm, 1.0 W cm^-2^, 10 min | 1064 nm, 1.0 W cm^-2^, 10 min | [29] |
| Gd/CuS@PEI-FA-PS NGs | FA | KB-LFAR, KB-HFAR | 26.7 | 1064 nm, 0.6 W cm^-2^, 5 min | 1064 nm, 0.6 W cm^-2^, 10 min | [30] |
| ^99m^Tc-M-CuS-PEG | / | 4T1, CT26 | / | 1064 nm, 0.5 W cm^-2^, 8 min | 1064 nm, 0.5 W cm^-2^, 8 min | [31] |

**Table S2.** CuS-based PTT combined with chemotherapy.

| Names of CuS-based nanoplatforms | Tumor cell lines | Drugs | DEE (%) | DLC (%) | PCE (%) | Lasers used in vitro | Lasers used in vivo | Ref. |
| --- | --- | --- | --- | --- | --- | --- | --- | --- |
| CuS HNs | HepG2 | DOX | / | 2.69 | 30 | 1064 nm, 3.0 W cm^-2^ | 1064 nm, 3.0 W cm^-2^, 10 min | [32] |
| CuS-ZnS | HeLa, 4T1, MCF-7 | 5-Fu, PTX | 5-Fu: 97.63, PTX: 41.88 | / | 40.15 | 808 nm, 0.65 W cm^-2^, 5 min | 808 nm, 0.65 W cm^-2^, 5 min | [33] |
| Au-CuS YSNPs | 4T1 | DOX | 9.11 | / | 808 nm: 62.44, 980 nm: 61.34 | 808 nm, 980 nm, 0.75 W cm^-2^, 5 min | 808 nm, 980 nm, 0.75 W cm^-2^, 5 min | [40] |
| DCSNGs | HepG2, H22 | DOX | 71.9 | 14.7 | / | 808 nm, 0.5 W cm^-2^, 30 min | 808 nm, 1.0 W cm^-2^, 25 min | [41] |
| CuS@Fe-MOF-DOX | CT26 | DOX | 50.8 | 27.5 | 39.7 | 1064 nm, 1.0 W cm^-2^, 10 min | 1064 nm, 1.0 W cm^-2^, 10 min | [42] |
| CuS@BSA-HMONs-DOX | Saos-2, KB | DOX | / | 42.9 | 51.5 | 808 nm,1.0 W cm^-2^, 5 min | 808 nm, 1.0 W cm^-2^, 5 min | [44] |
| HMSNs-CS-DOX@CuS | MDA-MB-231 | DOX | / | 46.1 | 36.4 | 808 nm, 1.0 W cm^-2^, 5 min | 808 nm, 1.0 W cm^-2^, 10 min | [45] |
| YSPMOs(DOX)@CuS | MDA-MB-231 | DOX | 89.4 | 38.6 | 70.8 | 980 nm, 1.0 W cm^-2^, 10 min | 980 nm, 1.0 W cm^-2^, 10 min | [46] |
| CuS_NC_ @DOX@MnO_2‑NS_ | HepG2 | DOX | 66 | / | 28 | 808 nm, 0.5 W cm^-2^, 5 min | 808 nm, 0.5 W cm^-2^, 3 min | [50] |
| M-Pt/PEG-CuS | SK-OV-3 | Cispt(IV) | / | 9.5 | 40.2 | 1064 nm, 1.5 W cm^-2^, 5 min | 1064 nm, 1.5 W cm^-2^, 5 min | [51] |
| CuS-DDTC NDs | 4T1 | DDTC | / | 19.5 | 56.5-60.9 | 808 nm, 1.5 W cm^-2^, 5 min | 808 nm, 1.5 W cm^-2^,5 min | [52] |
| BSD NPs | B16 | Cu(DTC)_2_ | / | 12.1-20.5 | 49.5 | 808 nm, 2.0 W cm^-2^, 10 min | 808 nm, 1.0 W cm^-2^, 5 min | [53] |
| MLNPs | 4T1 | Cu-meloxicam | / | 7.8 | 33.2 | 808 nm, 1.5 W cm^-2^, 5 min | 808 nm, 2.0 W cm^-2^, 5 min | [56] |
| BiOI@CuS | A549 | DOX | / | / | / | 980 nm, 1.5 W cm^-2^, 3 min | 980 nm; 1.5 W cm^-2^, 3 min | [57] |

**Table S3.** CuS-based PTT combined with PDT or CDT.

| Names of CuS-based nanoplatforms | Therapies | Tumor cell lines | Drugs | DLC (%) | PCE (%) | Lasers used in vitro | Lasers used in vivo | Ref. |
| --- | --- | --- | --- | --- | --- | --- | --- | --- |
| PEG-HMON@CuS/Gd | PDT | HGC-27 | / | / | 82.4 | 808 nm, 0.8 W cm^-2^, 5 min | 808 nm, 0.8 W cm^-2^, 8 min | [15] |
| CuS NCs | PDT | 4T1 | / | / | 46 | 808 nm, 2.0 W cm^-2^, 10 min | 808 nm, 2.0 W cm^-2^, 10 min | [68] |
| Cu-DhaTPh | PDT | HCT116 | / | / | 27.7 | 660 nm, 50 mW cm^-2^, 5 min; 808 nm, 2.0 W cm^-2^, 10 min | 660 nm, 50 mW cm^-2^, 5 min; 808 nm, 1.5 W cm^-2^,10 min | [69] |
| CuS@COF-BDP | PDT | MCF-7 | / | / | 42 | 520 nm, 50 mW cm^-2^, 5 min; 1064 nm, 1.5 W cm^-2^, 5 min | 520 nm, 50 mW cm^-2^, 10 min; 1064 nm, 1.1 W cm^-2^, 10 min | [70] |
| Ce6/CuS@Carbon | PDT | B16F10 | Ce6 | 13 | / | 660 nm, 2.0 W cm^-2^, 10 min | 660 nm, 2.0 W cm^-2^, 10 min | [73] |
| MSN/CuS/Ir/BSA | PDT | HeLa | / | / | 31.7 | 808 nm, 0.72 W cm^-2^, 5 min | 808 nm, 0.72 W cm^-2^, 5 min | [74] |
| UCNPs-Ce6@mSiO_2_-CuS | PDT | MCF-7, B16 | Ce6 | / | / | 980 nm, 4.0 W cm^-2^, 2 min | 980 nm, 4.0 W cm^-2^, 5 min | [76] |
| CuS-MnS_2_ | PDT | A2780, H460, A549 | / | / | 67.5 | 808 nm, 1.0 W cm^-2^, 3 min | 808 nm, 1.0 W cm^-2^, 10 min | [79] |
| CuS-NiS_2_ | PDT | AGS, MKN-45 | / | / | 52.2 | 808 nm, 1.0 W cm^-2^, 4 min | 808 nm, 1.0 W cm^-2^, 10 min | [80] |
| BSA-Ag:CuS | CDT | 4T1 | / | / | 37.8 | 1064 nm, 1.25 W cm^-2^, 10 min | 1064 nm, 1.25 W cm^-2^, 10 min | [81] |
| CuS@G5-PEG/GOD | CDT | 4T1 | GOx | / | / | 808 nm, 1.2 W cm^-2^, 5 min | 808 nm, 1.2 W cm^-2^, 5 min | [83] |
| HKUST-1 | CDT | HeLa, CT26 | / | / | 45.7 | 808 nm, 0.76 W cm^-2^, 5 min | 808 nm, 0.76 W cm^-2^, 5 min | [86] |

**Table S4.** CuS-based PTT combined with immunotherapy.

| Names of CuS-based nanoplatforms | Therapies | Tumor cell lines | Lasers used in vitro | Lasers used in vivo | Ref. |
| --- | --- | --- | --- | --- | --- |
| CuS@mSiO_2_-PFP-PEG | Immunotherapy | MDA-MB-231, 4T1 | 808 nm, 1.5 W cm^-2^, 4 min | 808 nm, 2.0 W cm^-2^ | [91] |
| CuS NPs-PEG-Mal | Immunotherapy | 4T1 | 808 nm, 2.0 W cm^-2^, 5 min | 808 nm, 0.45 W cm^-2^, 5 min | [92] |
| CSP@IL-12 | Immunotherapy | B16F10 | 1064 nm, 0.8 W cm^-2^, 10 min | 1064 nm, 0.6 W cm^-2^, 5 min | [93] |
| CuS-RNP@PEI | Immunotherapy | MDA-MB-231, A375, B16F10 | 808 nm, 2.1 W cm^-2^, 10 min | 808 nm, 2.1 W cm^-2^, 10 min | [94] |

**Table S5.** CuS-based PTT combined with two or more therapies.

| Names of CuS-based nanoplatforms | Target ligands | Therapies | Tumor cell lines | Drugs | DEE (%) | DLC (%) | PCE (%) | Lasers used in vitro | Lasers used in vivo | Ref. |
| --- | --- | --- | --- | --- | --- | --- | --- | --- | --- | --- |
| CuS@COFs-BSA-FA/DOX | FA | Chemo/CDT | 4T1 | DOX | / | 4.35 | 21.5 | 808 nm, 1.0 W cm^-2^, 8 min | 808 nm, 0.65 W cm^-2^, 8 min | [67] |
| MC-CHO/CMC/CuS/DOX@ZIF-8 | / | Chemo/PDT | 4T1 | DOX | 57.93 | 17.38 | / | 808 nm, 1.0 W cm^-2^ | 808 nm, 1.0 W cm^-2^, 5 min | [101] |
| Gox@CuS NPs | / | PDT/CDT | B16F10, MDA-MB-231 | GOx | / | / | / | 808 nm, 1.0 W cm^-2^, 10 min | 980 nm, 5.0 W cm^-2^, 10 min | [108] |
| NP-Cu | / | PDT/CDT | HepG2, HCT116 | Ce6, AQ4N | Ce6: 17.8, AQ4N: 13.2 | / | / | 660 nm, 100 mW cm^-2^, 1 min; 808 nm, 1.5 W cm^-2^, 10 min | 660 nm, 200 mW cm^-2^, 10 min; 808 nm, 3.0 W cm^-2^, 10 min | [111] |
